# TaWAKL8-2B, a wall-associated receptor-like kinase, mediates wheat rust resistance by linalool and ROS accumulation

**DOI:** 10.1007/s44154-025-00248-3

**Published:** 2025-08-18

**Authors:** Mengying He, Shan Zhang, Chunlei Tang, Yurong Yan, Zhongming Zhang, Jianfeng Wang, Ning Wang, Xiaojie Wang

**Affiliations:** State Key Laboratory for Crop Stress Resistance and High-Efficiency Production, College of Plant Protection, Northwest A&F University, Yangling, 712100 China

**Keywords:** Wall-associated receptor-like kinase, *Puccinia striiformis* f. sp. *tritici* (*Pst*), Wheat, Linalool, ROS

## Abstract

Wall-associated receptor kinases (WAKs) and WAK-likes (WAKLs) play pivotal roles in regulating plant immunity, through multiple downstream signaling components. However, knowledge of WAKs/WAKLs in wheat immune responses to rust diseases remain limited. In this study, we identified and characterized a wheat WAKL, *TaWAKL8-2B*, which is upregulated during wheat resistance to both *Puccinia striiformis* f. sp. *tritici* (*Pst*) and *Puccinia triticina* (*Ptt*), indicating its role in wheat resistance to these two rust fungi. Transgenic wheat plants overexpressing *TaWAKL8-2B* exhibited enhanced resistance to stripe rust and leaf rust, accompanied by increased reactive oxygen species (ROS) production and up-regulated defense-related gene expression. Whereas, knockout *TaWAKL8-2B* reduced resistance to *Pst* and *Ptt* with less ROS accumulation, highlighting its positive role in wheat resistance. RNA-seq analysis revealed that 33 genes encoding ROS-scavenging enzymes were upregulated in *TaWAKL8-2B*-KO plants, explaining the reduced ROS. KEGG analysis enriched the monoterpenoid pathway, particularly the linalool biosynthesis pathway, with linalool synthases significantly downregulated in *TaWAKL8-2B*-KO plants. Correspondingly, linalool synthase content and linalool content decreased in knockout plants. Collectively, our findings uncover a novel mechanism by which TaWAKL8-2B positively modulates wheat rust resistance through modulating linalool biosynthesis and peroxidase activity. These results enhance our understanding of TaWAKL8-2B mediated immune signaling and offer a promising gene for improving wheat broad-spectrum resistance to rust diseases.

**Supplementary Information:**

The online version contains supplementary material available at 10.1007/s44154-025-00248-3.

## Introduction

Plants have evolved an elaborate and sophisticated innate immune system to fend off pathogen invasions. The pathogen-associated molecular pattern (PAMP)-triggered immunity (PTI) is the first line of defense (Ngou et al. 2021). Upon perception of PAMPs, receptor-like kinases (RLKs) and receptor-like proteins (RLPs) on the plant cell surface, referred to as pattern recognition receptors (PRRs) are activated and transduce the signal to downstream immune components (Macho and Zipfel 2014; Boutrot and Zipfel 2017; DeFalco and Zipfel 2021). Wall-associated kinases and WAKs-like proteins constitute a plant-specific subfamily of receptor-like kinase superfamily. WAKs are characterized by a signal peptide at the amino (N) terminus, an extracellular WAK galacturonan-binding (GUB) domain, an epidermal growth factor (EGF)-like domain or EGF calcium-binding (EGF-Ca^2+^) domain, a transmembrane (TM) domain, and a carboxyl (C)-terminal cytosolic Ser/Thr protein kinase domain (Anderson et al. 2001; Verica and He 2002). The extracellular domain of WAKs facilitates binding to pectin and involved in cytoplasm-cell wall communication, whereas the intracellular kinase domain is involved in activating of cytoplasmic signaling cascades that regulate plant development and defense responses (Decreux and Messiaen 2005; Kohorn and Kohorn 2012; Kohorn 2015; Stephens et al. 2022).

An increasing number of studies highlight the critical role of WAKs in plant immune response against bacteria and fungi. AtWAK1, the first identified WAK gene in plants, binds with high affinity to pectin fragments or oligogalacturonides induced by pathogens and damage, playing a role in plant immunity (Wagner and Kohorn 2001; Decreux and Messiaen 2005; Brutus et al. 2010). AtWAK2 binds to pectin and activates mitogen-activated protein kinases (MPKs) in Arabidopsis (Kohorn et al. 2009). AtWAKL22 (also named as RFO1) has a dual function in pectin sensing during both plant development and defense response to *Fusarium* wilt disease (Diener and Ausubel 2005; Huerta et al. 2023). Immunity-related WAK/WAKL genes have been identified in other crops. In rice, *Xa4*, encoding a WAK, offers durable resistance against *Xanthomonas oryzae* by enhancing cellulose synthesis to fortify the plant cell wall (Hu et al. 2017). Other rice WAKs like OsWAK1, OsWAK14, OsWAK91, OsWAK92, OsWAK112d, OsWAK25 and OsWAKL21.2 are associated with resistance to various pathogens (Li et al. 2009; Delteil et al. 2016; Harkenrider et al. 2016; Malukani et al. 2020). In maize, phosphorylation transmission between ZmWAK-ZmSnRK1α2-ZmWRKY53 reduces ZmWRKY53 protein level, downregulating genes in nutrient transport and metabolism, thus starving pathogens of nutrient (Zhang et al. 2024). ZmWAK17 triggers cell death and mediates resistance to *Fusarium graminearum* (Zuo et al. 2022). In wheat, several WAK/WAKLs have been identified to participate in resistance to various pathogens. Wheat WAKL Stb6 confers resistance to fungal pathogen *Zymoseptoria tritici* (Saintenac et al. 2018). TaWAK6 is involved in wheat resistance to biotrophic leaf rust (Dmochowska-Boguta et al. 2020) and TaWAK-6D mediates broad resistance to *Fusarium pseudograminearum* and *Rhizoctonia cerealis* (Qi et al. 2021). TaWAK2A-800 provides resistance to *Fusarium* head blight and sharp eyespot (Guo et al. 2021). Conversely, the wheat WAK gene *Snn1* confers susceptibility to necrotrophic pathogens *Parastagonospora nodorum*, triggered upon recognition of the fungal effector SnTox1 (Liu et al. 2012; Shi et al. 2016). However, the mechanisms underlying the function of these genes remain to be explored.

Plants have evolved diverse mechanisms to defend against pathogens. The interaction of plants with pathogens induced production of phytoalexins, such as phenylpropanoids, alkaloids, or terpenoids (Ahuja et al. 2011; Polturak et al. 2022). ZmWAK-RLK1 (Htn1) confers quantitative resistance to northern corn leaf blight by regulating genes related to the biosynthesis of the secondary metabolites benzoxazinoids (Yang et al. 2019). In rice, transgenic plants overexpressing linalool synthase gene *OsLIS* showed enhanced resistance to *Xanthomonas oryzae* pv. *oryzae* (*Xoo*), presumably due to the up-regulation of defence-related genes (Taniguchi et al. 2014). The receptor-like kinase OsBDR1 interacts with MPK3 to negatively regulate rice blast resistance by suppressing the jasmonate (JA) and terpenoid pathways (Wang et al. 2023a). Linalool can induce hypersensitive reaction (HR), boost the expression of defense related enzymes, and increase the accumulation of H_2_O_2_ and salicylic acid (SA) content. This results in resistance against a wide variety of pathogens in tobacco plants (Jiang et al. 2023). Given its potential as an antiviral agent and plant immune activator, the function of linalool in wheat rust diseases deserves further study.

ROS is povotal in plant defense, and its homeostasis is tightly regulated by ROS-scavenging enzymes (Qi et al. 2017; Dumanović et al. 2021; Mittler et al. 2022; Wu et al. 2023). For instance, rice BSR-D1 promotes the expression of peroxidase, thereby suppressing immunity to a variety of *Magnaporthe oryzae* isolates (Li et al. 2017). The Ca^2+^ sensor ROD1 recruits catalase CatB to the plasma membrane, facilitating CatB-mediated ROS scavenging. This process degrades H_2_O_2_ and suppresses plant immunity (Gao et al. 2021). OsUMP1 regulates peroxidase/catalase-mediated immunity suppression, buffering the accumulation of H_2_O_2_ upon pathogen infection (Hu et al. 2023). In maize, ZmWAKL^Y^ initiates immune signaling via phosphorylation of ZmWIK, ZmBLK1, and ZmRBOH4, ultimately triggering a ROS burst and enhancing resistance to gray leaf spot (Zhong et al. 2024). In wheat, Yr36 phosphorylates the thylakoid-associated ascorbate peroxidase in chloroplast, conferring broad-spectrum resistance to stripe rust (Gou et al. 2015). Additionally, the CC-NBS-LRR protein TaRGA3 interacts with ascorbate peroxidase TaAPX6, leading to reduced enzyme activity and promoted ROS accumulation (Fang et al. 2024).

Wheat stripe rust and leaf rust pose a dire threat to global food security, causing substantial crop yield losses (Savary et al. 2019; Chen 2020). Planting resistant wheat cultivars is a cost-effective strategy to control rust diseases. Identifying novel molecular targets for effective and long-lasting resistance, and comprehensively understanding their molecular mechanisms, are essential for wheat resistance breeding (Khan et al. 2013; King et al. 2024). Given the important function of WAKs/WAKLs in plant immunity, wheat WAKs/WAKLs genes might be of great importance in wheat responses against rust disease and merit further investigation. Through expression pattern analyses of *Pst*-infected and *Ptt*-infected wheat leaves, we identified a WAKL gene *TaWAKL8-2B* that is up-regulated in response to *Pst* and *Ptt*. Genetic manipulation of *TaWAKL8-2B* demonstrated that it positively contributes to wheat resistance against both two rust fungi*.* RNA-seq results showed that knockout of *TaWAKL8-2B* led to downregulated expression of S-(+)-linalool synthase genes and upregulated ROS-scavenging enzymes. The results suggest that TaWAKL8-2B probably positively regulates S-(+)-linalool synthase and negatively modulates peroxidase to achieve the accumulation of linalool and ROS, thus endowing wheat with rust resistance. These findings present a promising gene target for improving wheat resistance to *Pst* and *Ptt* and offer new insights into the molecular mechanisms underlying rust fungi-wheat interactions.

## Results

### Wall-associated kinase TaWAKL8-2B involved in wheat-rust interaction

**Fig. 1:**
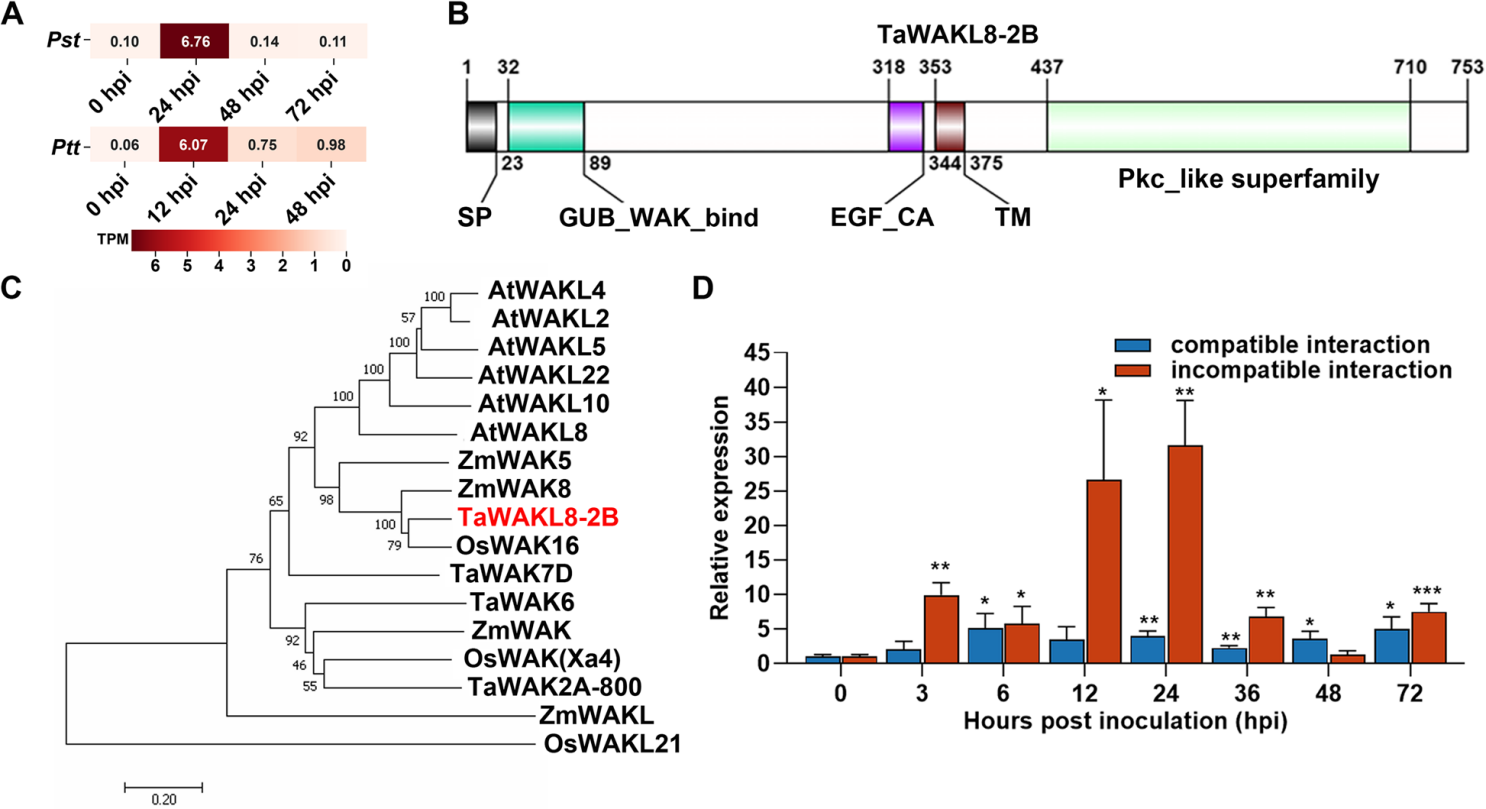
Identification and expression analysis of *TaWAKL8*-*2B* during the interaction of wheat with *Pst* and *Ptt*. **A** The expression pattern of *TaWAKL8-2B* during the interaction between wheat and *Pst* or *Ptt* by RNA-seq. TPM, Transcripts Per Kilobase of exon model per Million mapped reads. **B** Schematic diagram of the conserved domain of the TaWAKL8-2B protein. **C** Phylogenetic analysis of TaWAKL8-2B protein and other WAK/WAKL proteins. Phylogenetic tree was constructed by using the neighbor-joining phylogeny of MEGA 7.0. Bootstrap value is 1000. Ta, *Triticum aestivum*; Os, *Oryza sativa*; At, *Arabidopsis thaliana*; Zm, *Zea mays*. **D** Expression analysis of *TaWAKL8*-*2B* during wheat-*Pst* interaction by qRT-PCR. The second leaves of wheat cultivar Suwon11 were inoculated with urediospores of *Pst* race CYR23 (incompatible interaction) or *Pst* race CYR31 (compatible interaction) and sampled at different hours post-inoculation. The *TaEF-1α* served as an internal control. Error bars indicate the SD from three technical replicates. Experiments were independently repeated for three times with similar results. Asterisks indicate significant differences (Student’s *t* test, *p < 0.05, **p < 0.01, ***p < 0.001)

Transcriptome analysis revealed that *TaWAKL8-2B* is induced in wheat challenged by both *Pst* and *Ptt*, at 24 hpi and 12 hpi respectively (Fig. 1A), suggesting its involvement in wheat response to these two rust fungi. Full-length cDNA cloning revealed an open reading frame (ORF) of 2262 bp. TaWAKL8-2B protein consists of 753 amino acid residues with a pI of 7.66 and a calculated molecular mass of 82.92 kDa. TaWAKL8-2B contains a N-terminal signal peptide (SP), an extracellular galacturonan-binding (GUB) domain, which is hypothesized to be closely associated with the cell wall, a calcium-binding EGF domain, a transmembrane domain (TM) and an intracellular Ser/Thr protein kinase domain (Fig. 1B).

To decipher the evolutionary relationship of TaWAKL8-2B, we conducted a phylogenetic tree analysis incorporated TaWAKL8-2B and other WAKs/WAKLs from Arabidopsis, rice and maize. The results showed that TaWAKL8-2B grouped together with OsWAK16, ZmWAK5 and ZmWAK8 (Fig. 1C). Notably, TaWAKL8-2B is closely related to AtWAKL8 (Fig. 1C), an Arabidopsis gene implicated in stem secondary wall development, implying that TaWAKL8-2B may also participate in cell wall development.

We further investigated the expression pattern of *TaWAKL8-2B* during wheat-*Pst* interaction by qRT-PCR. In the incompatible interaction, the transcript level of *TaWAKL8-2B* started to increase as early as 3 hpi, peaking at 24 hpi with approximately a 30-fold increase compared to the initial level, and subsequently declined (Fig. 1D). In contrast, in the compatible interaction, the expression of *TaWAKL8-2B* remained at a relatively lower level throughout the process (Fig. 1D). These results suggest that TaWAKL8-2B may positively contribute to wheat resistance against *Pst* and could be involved in the early defense response to the fungal infection.

### TaWAKL8-2B functions as a positive regulator of wheat resistance to *Pst*

To elucidate the function of *TaWAKL8-2B* in wheat resistance to stripe rust, we employed CRISPR-Cas9-mediated gene editing to genetically inactivate *TaWAKL8-2B* in the wheat genome. Two guide RNAs (gRNAs) targeting the conserved regions in CDS of *TaWAKL8-2B* were designed using WheatCrispr. Two mutant line, *TaWAKL8-2B-*KO-L1 and *TaWAKL8-2B-*KO-L2 were obtained, in which CDS of *TaWAKL8-2B* contained one nucleotide insertions in the targeted region (Fig. S1).

**Fig. 2:**
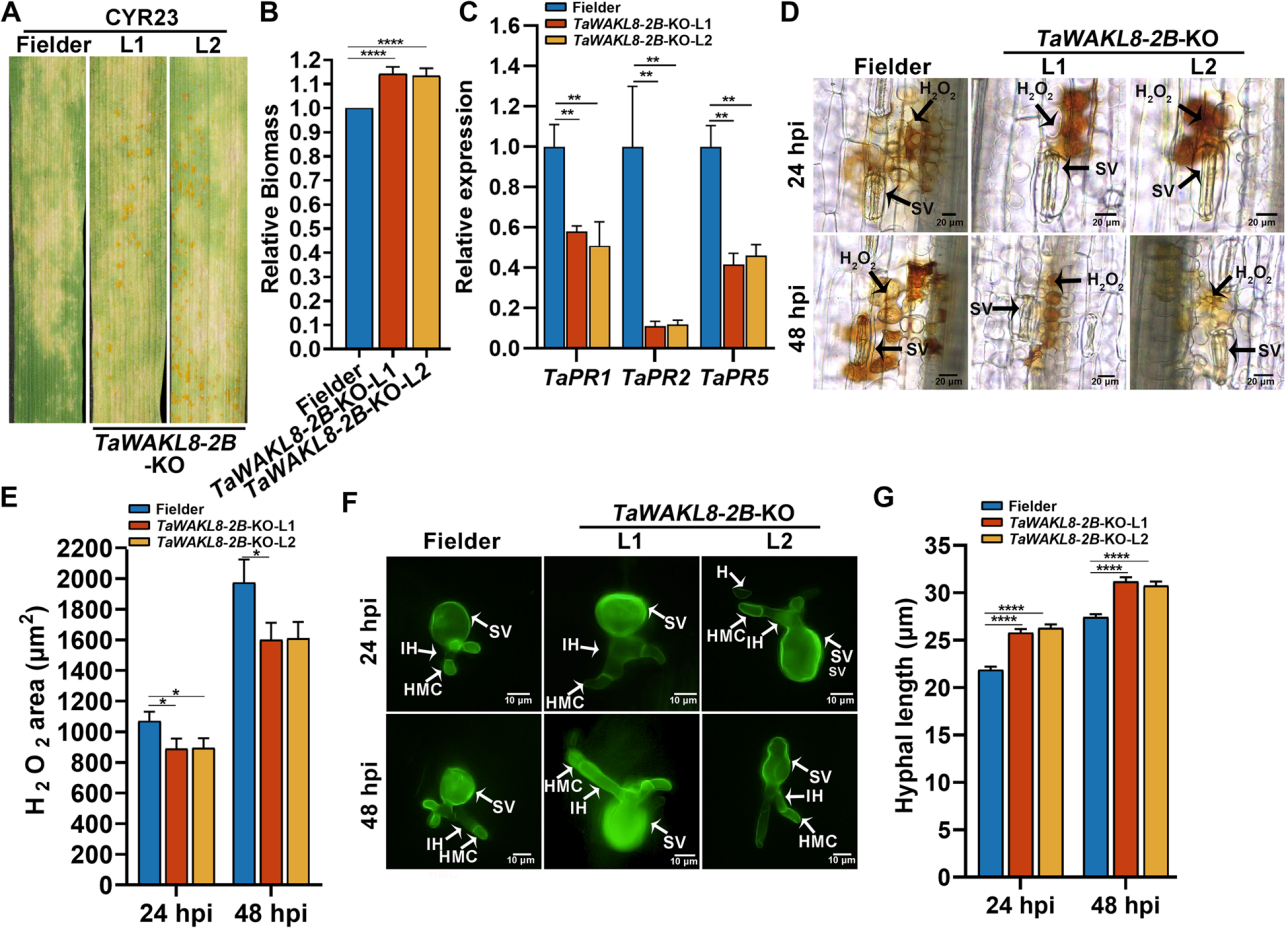
TaWAKL8-2B positively regulates wheat resistance to *Pst.* **A** Disease phenotype of *TaWAKL8*-*2B*-KO plants inoculated with *Pst* CYR23. Representative images of disease phenotype were taken at 14 dpi. **B** Relative fungal biomass in *TaWAKL8-2B*-KO plants. The relative biomass ratio (*Pst*/wheat) was assayed with DNA isolated from the leaves at 14 dpi. *TaEF-1α* and *PstEF* were used to normalize the DNA level of wheat and *Pst*, respectively. **C** Relative transcription levels of *TaPR1*, *TaPR2* and *TaPR5* in *TaWAKL8*-*2B*-KO plants inoculated with CYR23. **D** H_2_O_2_ accumulation at the infection sites in *TaWAKL8*-*2B*-KO plants stained with DAB at 24 and 48 hpi. **E** Quantification of tissue areas containing H_2_O_2_ from **D**. **F** Histological observation of *Pst* growth and development in *TaWAKL8*-*2B*-KO and Fielder plants. *Pst* infection structures were stained with wheat germ agglutinin conjugated to Alexa-488 and observed with an Olympus BX-53 microscope. SV, substomatal vesicle; IH, infection hypha; HMC, haustorial mother cell; H, haustorium; (g) Hyphal length was measured by Cellsens software. Values in (**E** and **G**) represent the mean ± SEM from three independent samples with 90 infection sites. Error bars in (**B** and **C**) indicate SD from three technical replicates. Experiments were independently repeated three times with similar results. Asterisks indicate significant differences (Student’s* t* test, *p < 0.05, **p < 0.01, ****p < 0.0001)

When compared to the Fielder plants inoculated with CYR23, which exhibited hypersensitive response (HR) symptoms, the *TaWAKL8-2B*-KO leaves showed alleviated HR response. Moreover, several fungal urediospores were observed surrounding the necrotic areas (Fig. 2A). To quantify the relative fungal biomass, we evaluated the ratio of fungal genomic DNA to wheat genomic DNA at 14 dpi. The result showed a significant increase in fungal biomass in *TaWAKL8-2B*-KO plants compared to Fielder plants (Fig. 2B). To determine whether the inactivation of *TaWAKL8-2B* affected the transcript accumulation of defense-related genes, we measured the transcript levels of three pathogenesis-related (PR) genes. The expression of *TaPR1*, *TaPR2* and *TaPR5* was significantly downregulated in *TaWAKL8-2B-*KO plants challenged by CYR23 (Fig. 2C). In addition, we used DAB and WGA staining to evaluate the accumulation of H_2_O_2_ around infection sites and conducted a histological observation of *Pst* growth. Remarkably, the production of H_2_O_2_ in *TaWAKL8-2B*-KO plants was significantly reduced compared to that in Fielder plants (Fig. 2D, E). Histological analysis showed that the hyphal length of *Pst* was longer in *TaWAKL8-2B*-KO plants at 24 and 48 hpi compared to the control (Fig. 2F, G), suggesting increased growth and development of the pathogen. Our results demonstrated that inhibiting the function of TaWAKL8-2B resulted in a reduction in H_2_O_2_ accumulation, which in turn promoted the growth and development of *Pst* during the infection process. Taken together, these findings consistently suggest that TaWAKL8-2B acts as a positive regulator of wheat resistance to *Pst*.

**Fig. 3:**
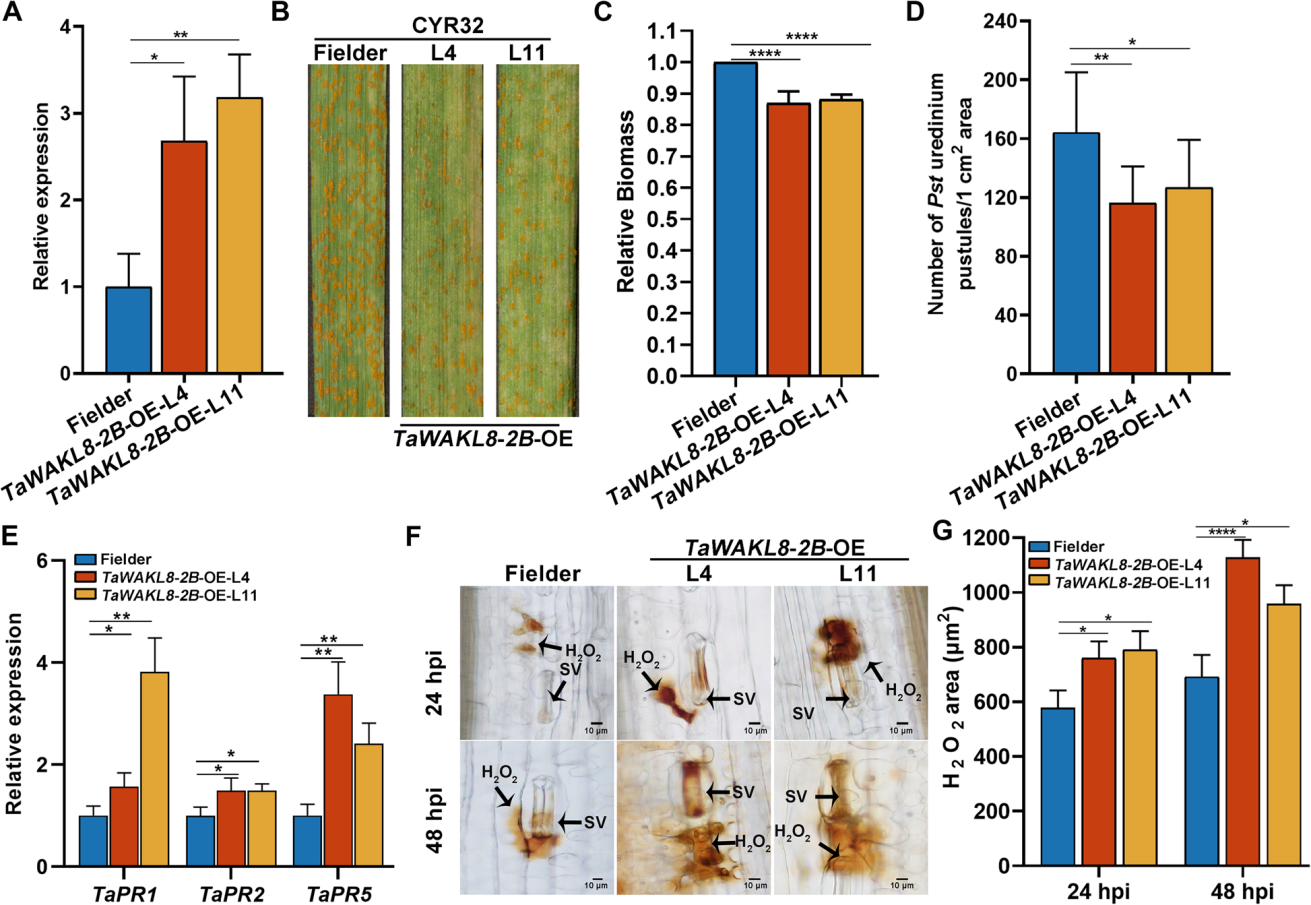
Overexpression of *TaWAKL8*-*2B* increases wheat stripe rust resistance. **A** Relative expression of *TaWAKL8-*2B in *TaWAKL8-2B*-OE lines. **B** Disease phenotypes of *TaWAKL8-2B*-OE plants inoculated with *Pst* CYR32 at 14 dpi. **C** Relative fungal biomass in *TaWAKL8-2B*-OE plants by qRT-PCR at 14 dpi. *TaEF-1α* and *PstEF* were used to normalize the DNA level of wheat and *Pst*, respectively. **D** Number of urediospore pustules per cm^2^ on the leaves from (B) counted using ImageJ. Error bars indicate the SD (*n* = 10). **E** Relative transcript levels of *TaPR1*, *TaPR2*, and *TaPR5* in *TaWAKL8*-*2B*-OE plants inoculated with *Pst* CYR32. **F** H_2_O_2_ accumulation at the infection sites in *TaWAKL8*-*2B*-OE stained with DAB at 24 and 48 hpi. **G** Quantification of tissue areas containing H_2_O_2_ from **F**. Values represent the mean ± SEM of three independent samples (*n* = 90). Error bars in (**A**, **C** and **E**) indicate the SD from three technical replicates. Experiments were independently repeated three times with similar results. Asterisks indicate significant differences (Student’s *t* test, *p < 0.05, **p < 0.01, ****p < 0.0001)

To further verify the positive role of *TaWAKL8-2B* in wheat resistance against *Pst*, we generated transgenic wheat lines overexpressing *TaWAKL8-2B* (*TaWAKL8-2B*-OE). In the T3 generation of transgenic lines, specifically *TaWAKL8-2B-*OE-L4 and *TaWAKL8-2B-*OE-L11, the *TaWAKL8*-*2B* transcript levels increased by 2.6-fold and 3.1-fold, respectively (Fig. 3A). Upon inoculation with the virulent *Pst* race CYR32, the wild-type Fielder plants developed numerous of urediospore pustules, whereas *TaWAKL8-2B-*OE-L4 and *TaWAKL8-2B-*OE-L11 plants displayed significantly enhanced resistance, characterized by a reduced number of urediospore pustules and the appearance of necrotic areas (Fig. 3B). Consequently, the fungal biomass in *TaWAKL8-2B-*OE plants was notably reduced compared to that in wild-type plants (Fig. 3C), and the number of urediospore pustules scored on *TaWAKL8-2B*-OE wheat plants was lower (Fig. 3D). Moreover, the expression of *TaPR1*, *TaPR2* and *TaPR5* was upregulated in *TaWAKL8-2B*-OE plants challenged by CYR32 (Fig. 3E). The area of H_2_O_2_ accumulation in *TaWAKL8-2B-*OE plants was significantly higher than that in Fielder at 24 and 48 hpi (Fig. 3F, G). Additionally, the hyphal length of *Pst* in *TaWAKL8-2B-*OE plants was shorter at 24 and 48 hpi (Fig. S2A, B). Collectively, these findings indicate that overexpressing *TaWAKL8-2B* increased wheat resistance to *Pst*.

### TaWAKL8-2B positively regulates wheat resistance to *Ptt*

**Fig. 4:**
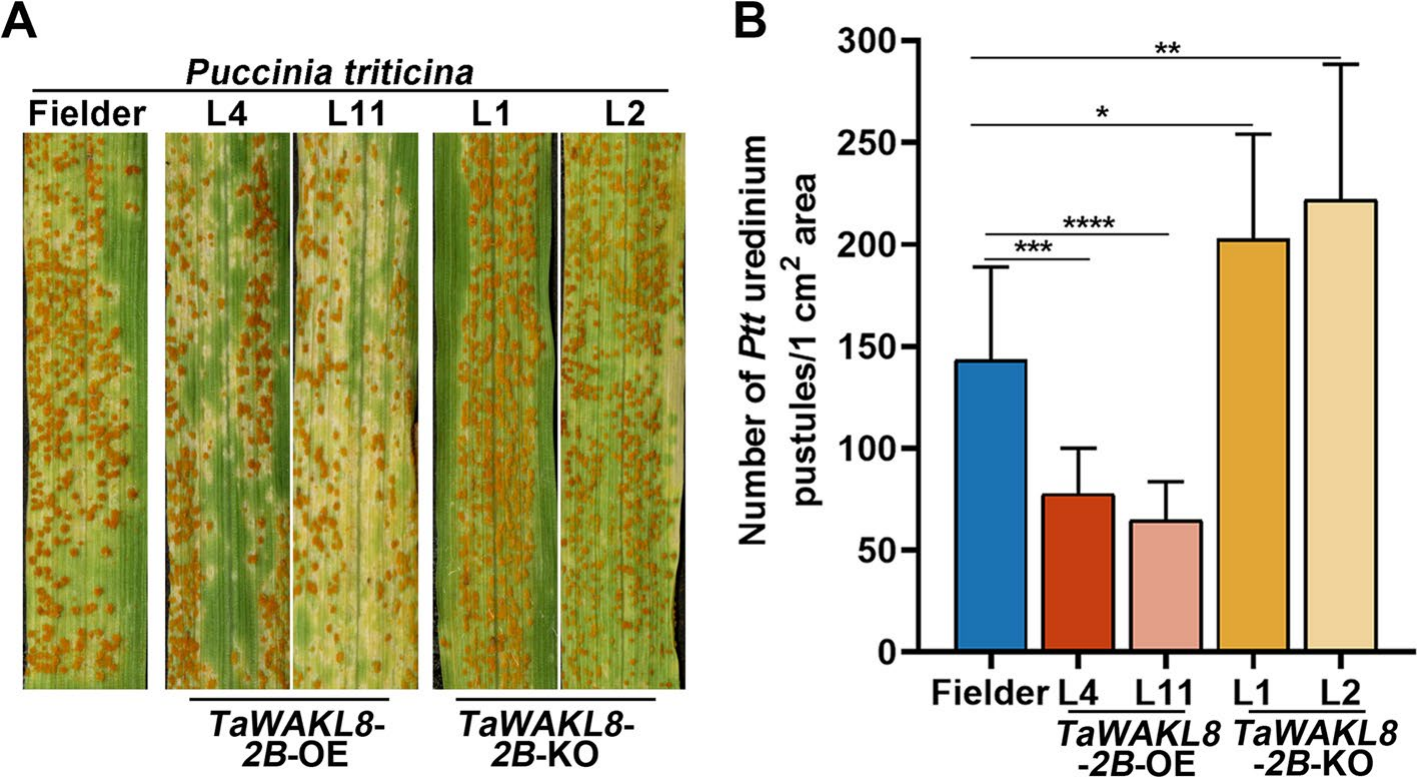
TaWAKL8-2B positively regulates wheat resistance to *Ptt*. **A** Disease phenotypes of *TaWAKL8*-*2B*-OE and *TaWAKL8*-*2B*-KO plants inoculated with *Ptt* at 10 dpi. **B** Number of *Ptt* pustules on the leaves from **A** counted using ImageJ. Error bars indicate the SD (*n* = 10). Asterisks indicate significant differences (Student’s *t* test, *p < 0.05, **p < 0.01, ***p < 0.001, ****p < 0.0001)

Based on the induced expression of *TaWAKL8-2B* by *Ptt* (Fig. 1A), we postulated its role in the interaction between wheat and *Ptt*. To explore whether *TaWAKL8-2B* contributes to wheat resistance against *Ptt*, we assessed the leaf rust resistance of *TaWAKL8*-*2B* knockout and overexpression lines. When inoculated with *Ptt*, *TaWAKL8-2B*-OE plants displayed significantly fewer urediospores compared to wild-type Fielder plants at 10 dpi. Conversely, knockout of *TaWAKL8-2B* resulted in an elevated number of urediospores (Fig. 4A). Quantitatively, the number of *Ptt* urediospores on *TaWAKL8-2B*-OE plants was significantly reduced, while in *TaWAKL8-2B*-KO plants, this number was significantly increased (Fig. 4B). The results provide evidence that *TaWAKL8-2B* acts as positive regulator wheat resistance to *Ptt*.

### *TaWAKL8-2B* knockout results in upregulation of ROS scavenging enzyme genes

**Fig. 5:**
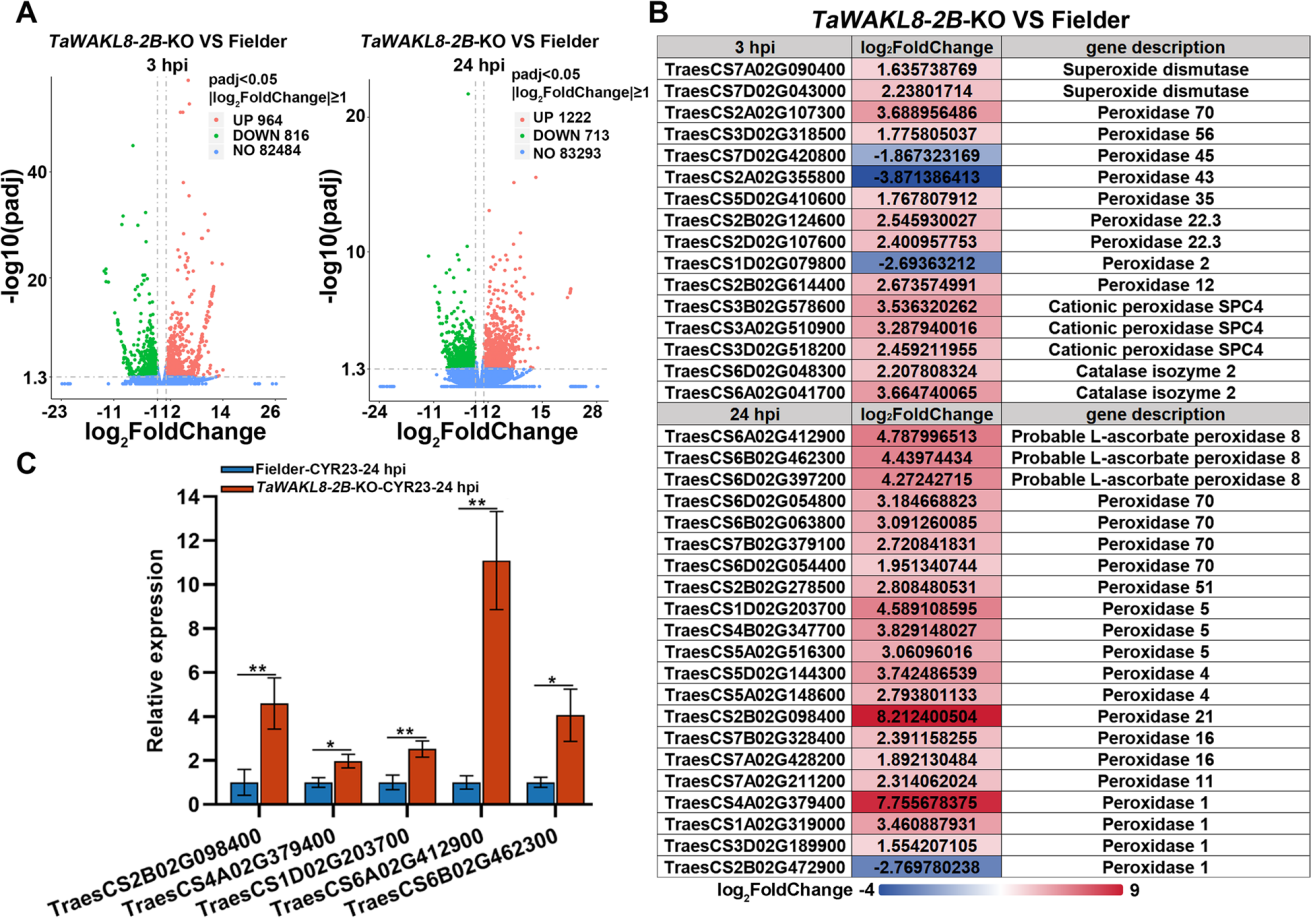
Differentially expressed genes in *TaWAKL8*-*2B*-KO transcriptome. **A** The vocano plot of differentially expressed genes in *TaWAKL8*-*2B*-KO vs Fielder at 3 hpi (left) and 24 hpi (right). Red dot, up-regulated DEGs; Green dot, down-regulated DEGs; Blue dot, genes with no significant difference in expression. **B** Differentially expressed genes associated with ROS scavenging in *TaWAKL8*-*2B*-KO vs Fielder DEGs. The indicated scale is the log_2_Foldchange value. **C** qRT-PCR analyses of the expression level of differentially expressed POD genes. Error bars indicate the SD from three technical replicates. Experiments were independently repeated three times with similar results. Asterisks indicate significant differences (Student’s *t* test, *p < 0.05, **p < 0.01)

To elucidate how TaWAKL8-2B responds to *Pst*, we carried out transcriptome analyses on *TaWAKL8-2B*-KO and wild-type Fielder plants at 3 hpi and 24 hpi with *Pst* race CYR23. 1780 and 1935 differentially expressed genes (DEGs) were identified at 3 hpi and 24 hpi, respectively (Table S2). At 3 hpi, among the DEGs, there were 964 up-regulated genes and 816 down-regulated genes. At 24 hpi, the DEGs included 1222 up-regulated genes and 713 down-regulated genes (Fig. 5A). Notably, we identified 37 ROS scavenging enzyme genes among the DEGs, and most of them were peroxidases (Fig. 5B). Except for 4 down-regulated peroxidase genes, the remaining 33 ROS scavenging enzyme genes were all up-regulated (Fig. 5B). Peroxidase (POD) enzymes are well-recognized for their essential roles in plant immunity through the scavenging of ROS (Almagro et al. 2009; Mittler et al. 2022). We selected the top 5 POD genes for further validation using qRT-PCR. The results showed that the expression of these genes was up-regulated in *TaWAKL8-2B*-KO plants (Fig. 5C), which is consistent with the reduced ROS accumulation observed by DAB staining (Fig. 2D, E). These findings suggest that the regulation of ROS by ROS scavenging enzyme genes may be an integral part of the resistance mechanism mediated by TaWAKL8-2B.

### TaWAKL8-2B-mediated resistance is associated with linalool biosynthesis

**Fig. 6:**
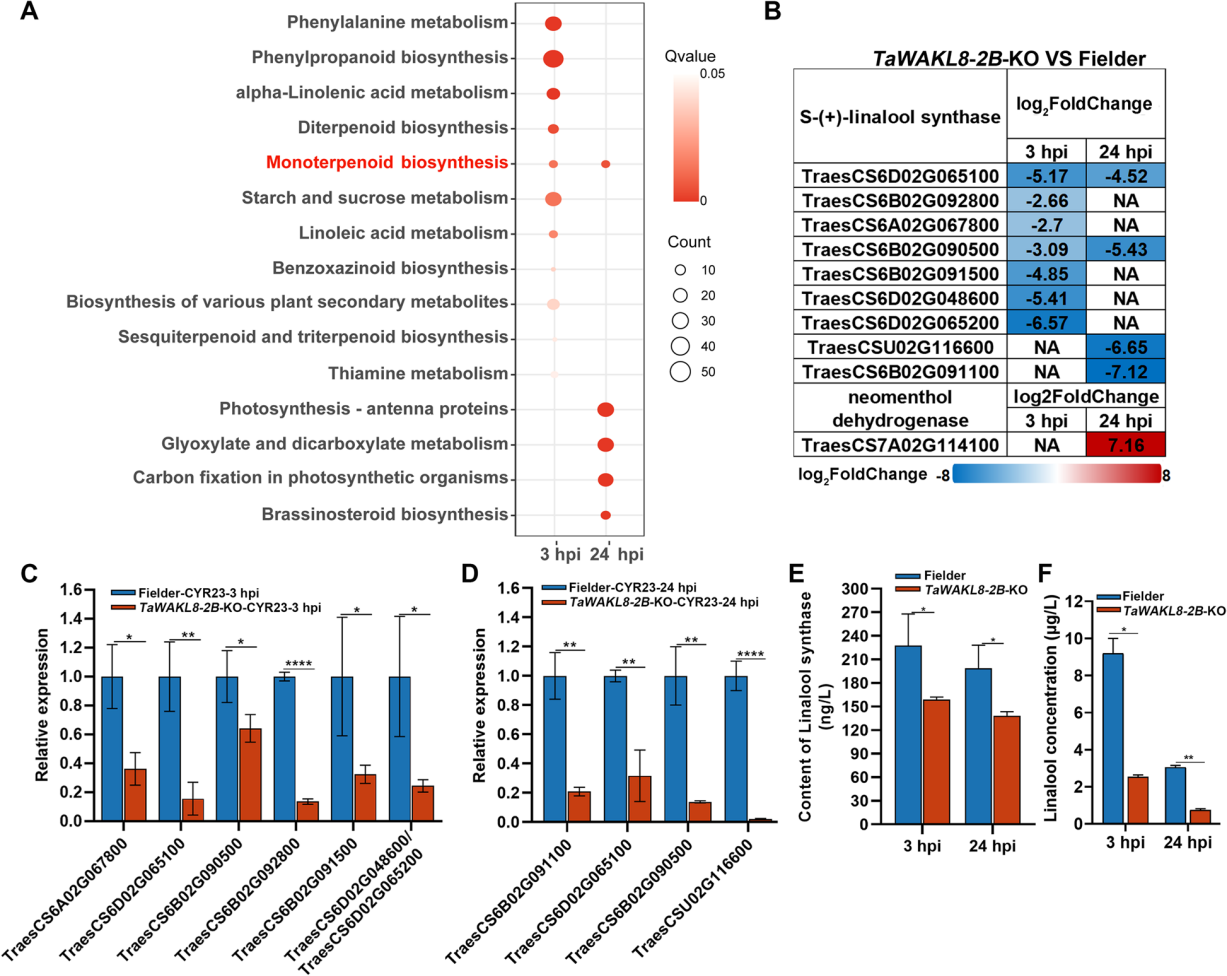
Enriched monoterpenoid pathway in *TaWAKL8-2B*-KO plants by KEGG analysis. **A** KEGG enrichment analysis of DEGs in *TaWAKL8-2B*-KO plants. **B** Expression pattern of DEGs involved in the monoterpenoid pathway. The indicated scale is the log_2_Foldchange value. **C** and **D** qRT-PCR analysis of differentially expressed S-(+)-linalool synthase genes in the *TaWAKL8*-*2B*-KO plants at 3 hpi (**C**) and 24 hpi (**D**). **E** Content of linalool synthase in *TaWAKL8-2B*-KO and Fielder plants measured by Elisa. Error bars indicate the SD from three technical replicates. **F** Content of linalool in *TaWAKL8-2B*-KO and Fielder plants measured by GC–MS. Error bars indicate the SD from two biological replicates. Asterisks indicate significant differences (Student’s *t* test, *p < 0.05, **p < 0.01, ****p < 0.0001)

Furthermore, Kyoto Encyclopedia of Genes and Genomes (KEGG) enrichment analysis of DEGs pointed out another important clue of TaWAKL8*-*2B-mediated immune responses. DEGs in 3 hpi were significantly enriched in the biosynthesis pathway of various plant primary and secondary metabolites (Fig. 6A). And the DEGs at 24 hpi was enriched in pathways related to photosynthesis, glyoxylate and dicarboxylate metabolism, brassinosteroid biosynthesis and monoterpenoid biosynthesis (Fig. 6A).

Moreover, at 3 hpi, the majority of DEGs involved in linolenic acid metabolism, starch and sucrose metabolism, benzoxazinoid biosynthesis, biosynthesis of various plant secondary metabolites, thiamine metabolism and terpenoids biosynthesis were down-regulated in *TaWAKL8-2B*-KO plants (Fig. S3A, Table S3). At 24 hpi, according to the KEGG analysis, most of the genes in the monoterpenoid biosynthesis and brassinosteroid biosynthesis showed down-regulation (Fig. S3B, Table S3).

Monoterpenoid, diterpenoid, sesquiterpenoid, triterpenoid and benzoxazinoid are pivotal secondary metabolites that exert significant impacts on plant immunity (Ahuja et al. 2012). Based on these findings, we hypothesized that the regulation of secondary metabolites-related genes might be an essential factor for TaWAKL8-2B-mediated immune response. We then focused on a specific down-regulated monoterpenoid pathway, which was enriched at both 3 hpi and 24 hpi (Fig. 6A). All nine S-(+)-linalool synthase genes (LLSs) in the monoterpenoid pathway exhibited varying degrees of decrease in *TaWAKL8-2B*-KO plants (Fig. 6B). These genes were further validated using qRT-PCR, and the results were generally consistent with the RNA-seq data (Fig. 6C, D). Subsequently, we employed an enzyme-linked immunosorbent assay (ELISA) to test the linalool synthase content. The results revealed that the leaves of *TaWAKL8-2B*-KO plants had a lower linalool synthase content compared to those of the wild-type Fielder plants (Fig. 6E). S-(+)-linalool synthase plays a vital role in linalool biosynthesis (Chen et al. 2011; Wang et al. 2023b; Liu et al. 2024). The reduction in linalool synthase might lead to decreased linalool levels, thus we measured the content of linalool in wheat by GC–MS. Compared with Fielder plants, the linalool content in *TaWAKL8-2B*-KO plants decreased significantly at 3 hpi and 24 hpi (Fig. 6F). Therefore, we propose that the monoterpenoid pathway, particularly linalool, may be involved in *TaWAKL8-2B*-mediated wheat defense against *Pst*.

In summary, our findings indicate that TaWAKL8-2B plays a regulatory role in the expression of S-(+)-linalool synthase and peroxidase genes. By doing so, it increases the content of linalool and modulates the levels of ROS, both of which are integral to wheat defense mechanism against *Pst*. However, the immune signaling pathways by which TaWAKL8-2B regulates linalool biosynthesis and ROS scavenging require further exploration.

## Discussion

**Fig. 7:**
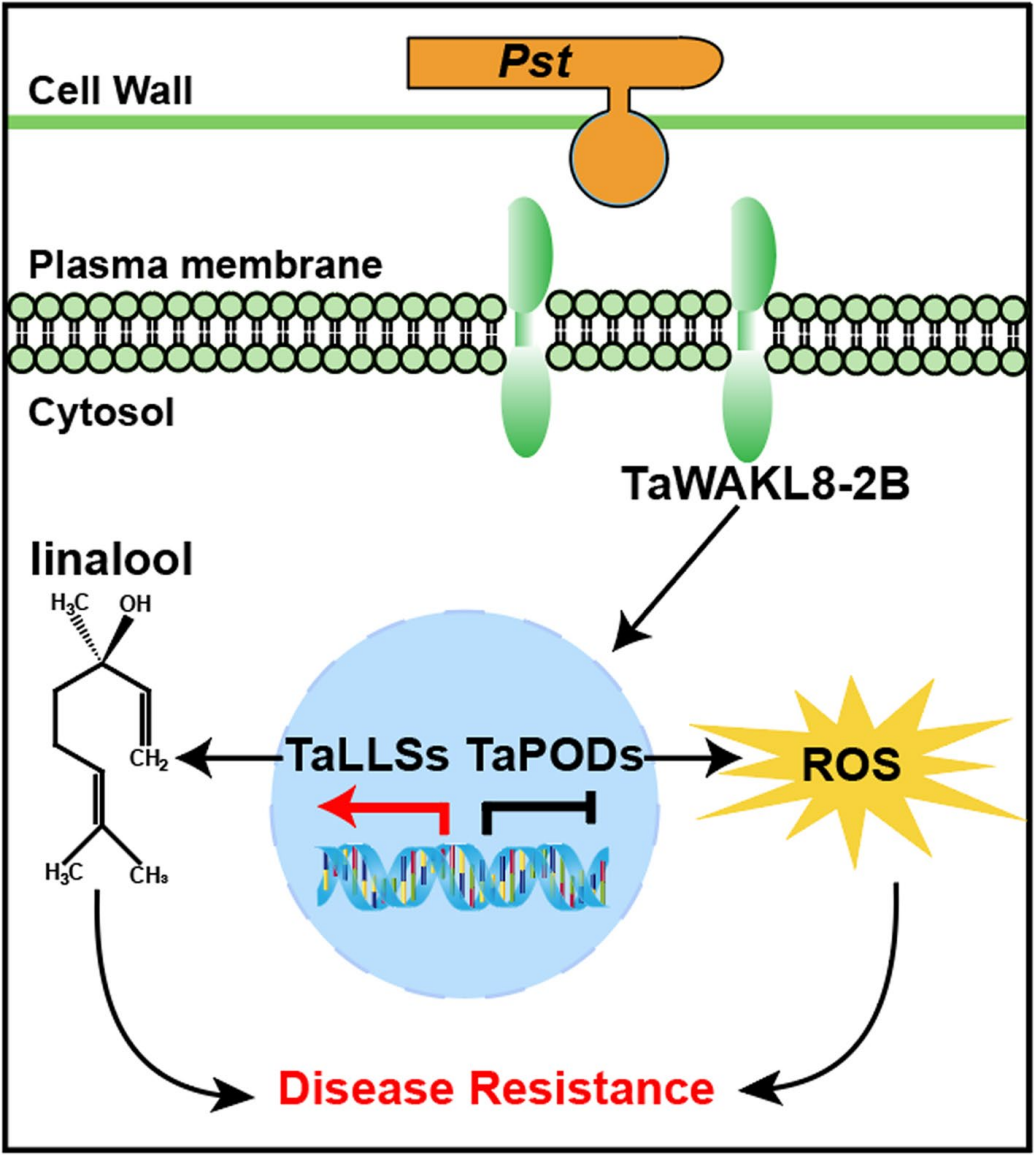
The proposed work model of TaWAKL8-2B-mediated wheat resistance to *Pst*. Upon *Pst* infection, *TaWAKL8*-*2B* is significantly upregulated. It positively regulates linalool synthase genes (*TaLLSs*), boosting the biosynthesis and accumulation of linalool, a key secondary metabolite for plant immunity. Simultaneously, it negatively regulates peroxidase genes (*TaPODs*) to increase ROS accumulation. These dual regulatory mechanisms endow wheat with resistance against *Pst* infection

Emerging evidences link WAKs/WAKLs to plant defense responses, yet their molecular mechanisms remain relatively unclear. In this study, we found that *TaWAKL8*-*2B* is induced by both *Pst* and *Ptt*, and confers broad-spectrum resistance to these biotrophic rust fungi. RNA-seq analysis of *TaWAKL8-2B-*KO revealed the important role of the monoterpenoid, especially linalool biosynthesis pathway, and peroxidase in wheat defense against rust. Based on these findings, we propose a model depicting the molecular mechanism by which TaWAKL8-2B contributes to wheat rust resistance (Fig. 7). During *Pst* infection, *TaWAKL8-2B* is significantly induced and positively regulates the expression of linalool synthase genes (*TaLLSs*) while negatively regulating peroxidase genes (*TaPODs*), thereby promoting the accumulation of linalool and ROS to confer resistance to *Pst* in wheat.

Several WAKs/WAKLs have been characterized to confer broad-spectrum disease resistance (Stephens et al. 2022). AtWAKL22 is important for resistance to *Fusarium oxysporum* and *Verticillium spp*. wilts (Diener and Ausubel 2005; Huerta et al. 2023). OsWAK25 and OsWAK91 contribute to rice resistance to the hemibiotrophic pathogens, *Xanthomonas oryzae* pv. *oryzae* and *Magnaporthe oryzae* (Harkenrider et al. 2016; Delteil et al. 2016; Huluka and Kumsa 2022). Notably, OsWAK25 has opposite effects on necrotrophic fungal pathogens, *Rhizoctonia solani* and *Cochliobolus miyabeanus* (Harkenrider et al. 2016), highlighting the diverse roles of WAKs in plant resistance to different types of pathogens. In wheat, two WAKs have been reported to regulate resistance to multiple pathogens. TaWAK-6D regulates resistance to *Fusarium* crown rot and sharp eyespot via modulating the expression of defense-related genes and might be involved in pectin-induced innate immune responses (Qi et al. 2021). Silencing of *TaWAK2A-800* compromised wheat resistance to both *Fusarium* head blight and sharp eyespot, possibly through a chitin-induced pathway (Guo et al. 2021). However, the broad-spectrum function of TaWAK-6D and TaWAK2A-800 require further validation using transgenic technology. In this study, through disease resistance evaluation of *TaWAKL8-2B* transgenic plants, we demonstrated that TaWAKL8-2B confers resistance against two crucial biotrophic fungi, *Pst* and *Ptt*. WAK/WAKLs equip plants with sophisticated surveillance capabilities, enabling them to detect PAMPs to initiate downstream plant immune response (Stephens et al. 2022). *TaWAKL8-2B*-OE line showed enhanced resistance to virulent *Pst* race CYR32, indicating its regulation role in basal resistance. Compromised resistance of TaWAKL8-2B-KO lines to avirulent *Pst* CYR23 implies ETI involvement, though PTI functionality remains plausible. TaWAKL8-2B likely serves as a convergence point for PTI and ETI signaling. While cross-pathogen resistance needs verification, it represents a prime candidate for developing broad-spectrum disease resistance in wheat.

Through evolutionary adaption, plants deploy terpenoid phytoalexins as chemical defenses against pathogens, with their biosynthesis orchestrated by terpene synthase genes (Chen et al. 2011; Abbas et al. 2017; Polturak et al. 2022). Mounting evidence highlights the dual roles of terpenoids in plant immunity, direct antimicrobial activity and defense signaling. Enhanced linalool production by ectopic expression of linalool synthase in rice improves resistance to *Xoo*, while the OsBDR1-OsMPK3 cascade coordinates terpenoid-mediated blast resistance (Taniguchi et al. 2014; Wang et al. 2023a). Volatile terpenoids like linalool and β-caryophyllene additionally prime systemic defenses in cereals (Li et al. 2018; Laupheimer et al. 2024). Our study reveals the involvement of *TaWAKL8-2B* in terpenoid-regulated immunity. Comparative transcriptomics demonstrated persistent downregulation of monoterpenoid biosynthesis in *TaWAKL8-2B*-KO plants, particularly affecting nine S-(+)-linalool synthase genes and corresponding linalool reduction (Fig. 6). This implicates TaWAKL8-2B in modulating linalool metabolism during pathogen response, though mechanistic details remain unclear.

Plants deploy diterpenoid, benzoxazinoid, and triterpenoid phytoalexins as specialized antimicrobials, with species-specific biosynthetic strategies. Rice DGC7 cluster genes (*OsCYP71Z2*/*OsTPS28*) produce antifungal diterpenoids (Li et al. 2013; Lu et al. 2018; Zhan et al. 2020; Liang et al. 2021). Maize ZmWAK-RLK1 regulates benzoxazinoids synthesis to confer quantitative resistance to northern corn leaf blight (Yang et al. 2019). Wheat maintains pathogen-inducible terpenoid clusters for broad-spectrum defense (Polturak et al. 2022). Our study positions TaWAKL8-2B as a multi-terpenoid regulator in wheat immunity. Transcriptome profiling revealed *TaWAKL8-2B*-KO not only suppresses monoterpenoid biosynthesis but dysregulates other terpenoid pathways, suggesting its central role in phytochemical defense coordination. This multi-terpenoid dysregulation highlights two critical knowledge gaps: 1) The quantitative relationship between TaWAKL8-2B activity and terpenoid phytoalexin profiles during rust infection, and 2) The signaling crosstalk coordinating different terpenoid pathways in wheat immunity. Integrated metabolomics-pathway networking analysis will decipher TaWAKL8-2B's regulatory hierarchy in plant-pathogen chemical warfare.

WAK proteins orchestrate redox homeostasis through dual control of ROS generation and scavenging across plant species. Canonical WAK-mediated immunity in wheat (Stb6), rice (OsWAK91), and maize (ZmWAKL^Y^) involves pathogen-induced ROS production (Delteil et al. 2016; Saintenac et al. 2018; Huluka and Kumsa 2022; Zhong et al. 2024). WAKs fine-tune ROS scavengers to maintain defense-optimized redox states. Rice *Bsr-d1*/wheat *Yr36*/cotton *GbWAKL14/* citrus *CsWAKL08* fine-tune peroxidases to inhibit ROS scavenging, achieving the defense-optimized redox balance for blast/stripe rust/vascular wilt/canker pathogens resistance (Gou et al. 2015; Li et al. 2017, 2020; Zhao et al. 2024). Our findings establish TaWAKL8-2B as a redox regulator in wheat-*Pst* interactions. Transcriptional upregulation of peroxidase (POD) genes in *TaWAKL8-2B*-KO plants (Fig. 5B-C), coupled with diminished ROS accumulation (Fig. 2E-F), demonstrates its role in suppressing ROS-scavenging machinery to maintain defense-effective oxidative bursts. This redox regulation operates in parallel with TaWAKL8-2B's control of linalool biosynthesis, revealing a dual chemical defense strategy combining antimicrobial terpenoids with oxidative stress.

## Conclusion

In this study, we characterized *TaWAKL8-2B*, a wall-associated kinase (WAK) gene in wheat, as a positive regulator of plant immunity against biotrophic rust fungi. Functional analysis revealed that TaWAKL8-2B enhances wheat resistance by coordinating the transcriptional regulation of S-(+)-linalool synthase and peroxidase-encoding genes, thereby promoting the accumulation of linalool and amplifying ROS bursts. Our findings highlight an evolutionarily conserved role of WAK family proteins in fungal pathogen defense and establish TaWAKL8-2B as a promising genetic resource for developing disease-resistant wheat cultivars through molecular breeding strategies.

## Methods

### Plant materials and inoculation assay

The wheat cultivar Suwon 11 (Su11) and Fielder exhibit resistance to *Puccinia striiformis* f. sp. *tritici* race CYR23 but are susceptible to race CYR32. The wheat cultivar Mingxian169 was used to propagate CYR23. Urediospores of *Pst* race CYR23 and race CYR32 were grown on Mingxian169 leaves and Su11 leaves respectively. Fresh urediospores were collected for inoculation experiment. Fielder was chosen as the background for generating transgenic plants. Wheat plants were grown in an incubator maintained at 16 °C with 85% relative humidity, under a 16-h light/8-h dark cycle. The inoculated plants were kept at 16℃ and 100% relative humidity for 24 h, and then returned to normal conditions as described above.

The urediospores of *Puccinia triticina* race THTT were grown on wheat cultivar Mingxian169 leaves, and the fresh urediospores were collected for inoculation assay. Fielder and transgenic plants were inoculated with the *Ptt* according to previously description (Liu et al. 2013). The inoculated seedlings were cultivated in the dark at 18 °C and 100% relative humidity for 24 h and then returned to normal growth conditions.

### Cloning and sequence analysis

For cloning, specific primers (Table S1) were designed according to the sequences in EnsembPlants, full-length sequence was obtained through PCR amplification from Su11 cDNA. To gain insights into the transcript expression pattern of *TaWAKL8-2B*, we queried data from Wheat expVIP (https://www.wheat-expression.com) and WheatOmics 1.0 (http://202.194.139.32). The expression data were subsequently visualized using ChiPlot (https://www.chiplot.online). Further analysis of the TaWAKL8-2B protein sequence was conducted using the NCBI Conserved Domain Database (https://www.ncbi.nlm.nih.gov/cdd) to identify conserved domains, and DeepTMHMM (https://services.healthtech.dtu.dk/services/DeepTMHMM-1.0/) to predict transmembrane helix regions.

### qRT-PCR analysis of gene expression

The second leaves of Su11 seedlings were inoculated with urediospores of CYR23 and CYR31 respectively, and the inoculated wheat plants were moisturized in the dark at 16℃ for 24 h and then move to an incubator at normal growth condition. Leaves were sampled at 0, 3, 6, 12, 24, 36, 48, and 72 h post inoculation (hpi) for RNA extraction. Samples were were snap-frozen in liquid nitrogen and RNA was extracted using Quick RNA isolation Kit (Huayueyang Biotech, Beijing, China) according to the manufacturer’s instructions. cDNA was synthesized utilizing the RevertAid First Strand cDNA Synthesis Kit (Thermo Fisher Scientific, Waltham, USA). qRT-PCR was conducted using the CFX96 Connect Real-Time PCR Detection System (Bio-Rad, Hercules, USA)) with ChamQ SYBR qPCR Master Mix (Vazyme Biotech, Nanjing, China), adhering the manufacturer’s protocols. The primer sequences are provided in Table S1. *TaEF-1α* (encoding wheat elongation factor, GenBank No. Q03033) served as an internal reference gene. The data were analyzed employing the comparative 2^–ΔΔCt^ method (Pfaffl 2001). The experiments were performed with three biological replicates, and representative results are presented.

### Generation and identification of wheat transgenic lines

To construct the overexpression vector, the full-length coding sequence of *TaWAKL8-2B* fused with an HA tag was cloned into the pANIC6E vector using Gateway cloning technology. The recombinant plasmid was transformed into *Agrobacterium tumefaciens* strain EHA105. Transgenic wheat lines were generated by *Agrobacterium*-mediated transformation of immature wheat embryos as described previously (Ishida et al. 2015). Genomic DNA was extracted for the regenerated plants for PCR analysis to confirm the integration of the transgene. Positive transgenic seedlings were further verified by qRT-PCR to assess the transcript level of *TaWAKL8-2B*.

For CRISPR-Cas9-mediated editing of *TaWAKL8-2B*, two sgRNAs were designed by WheatCrispr (https://crispr.bioinfo.nrc.ca/WheatCrispr/). The correct sgRNA1/2-Cas9-PCL4 recombinant construct was transformed into EHA105. CRISPR-Cas9 transgenic wheat lines were generated as described above. Genomic DNA was extracted for PCR detection of bialaphos resistance gene (bar) and sequencing using Hi-TOM system (Liu et al. 2019) to identify mutations in the *TaWAKL8-2B* gene. All primers for vector construction and detection are shown in Table S1.

### Histology analysis of *Pst* development and host defense response

Transgenic wheat and Fielder seedlings were grown and inoculated with *Pst* as described above. Leaf samples were collected at 24 and 48 h post-inoculation (hpi) and stained by 1.5 mg·mL^−1^ 3,3’-diaminobezidine (DAB, Sigma-Aldrich, California, USA) to detect H_2_O_2_ according to previously described methods (Wang et al. 2007). The H_2_O_2_ accumulation at infection area were visualized and quantified by Olympus BX-53 microscope (Olympus, Tokyo, Japan).

*Pst* was stained with wheat germ agglutinin conjugated to Alexa-488 (WGA) (Amresco, Solon, USA) as previously described (Xu et al. 2019). The hyphal length of *Pst* was observed and measured with Olympus BX-53 epifluorescence microscope (Olympus, Tokyo, Japan).

### Biomass assays

Wheat DNA and *Pst* DNA was extracted and diluted into a serial concentration for generation of the standard curves. *TaEF-1α* primers and *Pst*-*EF* primers were used for qRT-PCR analysis. Genomic DNA of the samples were extracted from the infected leaf tissue at 14 days post-inoculation (dpi) to determine the relative biomass (*Pst*/wheat ratio). Quantitative PCR was performed using a CFX96 Connect Real-Time PCR Detection System (Bio-Rad, Hercules, USA) to determine the *Pst* DNA content in the infected wheat leaves. The experiment includes three independently biological repeats.

### RNA-seq analysis of transgenic wheat

Twenty-day-old *TaWAKL8-2B*-KO transgenic and Fielder plants were used for transcriptome analysis. Three biological replicates of leaves were collected at 0, 3 and 24 hpi, and subjected to RNA extraction, RNA sequencing library construction, and the library preparations were sequenced on an Illumina Novaseq platform and 150 bp paired-end reads were generated by Novogene company. Clean reads were mapped to a reference wheat genome (https://ftp.ebi.ac.uk/ensemblge-nomes/pub/release-59/plants/fasta/triticum_aestivum/dna/). Differential expression gene analysis was performed using the DESeq2R package (1.20.0). Genes with an adjusted *P*-value ≤ 0.05 and log_2_foldchange ≥ 1 found by DESeq2 were assigned as differentially expressed genes (DEGs). KEGG enrichment analysis of differentially expressed genes was implemented by the clusterProfiler R package.

### Content of linalool synthase detection

The contents of linalool synthase were quantified by using the Plant linalool synthase ELISA kit (Fankew, Shanghai, China), following the manufacturer's instructions. One gram of fresh leaves was used to extract for analysis linalool synthase content. The optical density (OD) at 450 nm was then measured by a Multiskan Spectrum plate reader (Tecan, Männerdorf, Switzerland) to determine the enzyme concentration.

### Linalool detection by GC–MS

The content of linalool was detected by headspace solid-phase microextraction method. Grind 3 g fresh wheat leaves with 2 mL of water to obtain the wheat homogenate. Add 2 mL wheat homogenate to a solid phase extraction column (Agela, PE1506) and follow the instructions for impurity removal and concentration. The eluted liquid was vacuum-dried and then redissolved with 10mL citric acid phosphate buffer. Subsequently, 200 μL of glycosidase AR2000 was added and incubated at 40 °C for 16 h. Add 5 mL extracting solution, 1 g NaCl and 10 μL 4-methyl-2-pentanol (1.0083 g/L, Sigma-Aldrich, the internal standard) to a 10 mL injection flask. After shaking and heating at 40 °C for 30 min, headspace extraction was carried out for 30 min, and then the sample was detected by GC_MS (Thermo, ISQ7610). The temperature of the injection port was 250℃, and the carrier gas flow rate (helium, > 99.999%) was 1 mL/min. MS interface and ionic source temperatures were 250℃ and 280℃, respectively. The variation mode of column temperature was as follows: 45 ℃ for 3 min, raising to 180℃ at 6 ℃/min, and holding for 5 min. Electron impact spectra at 70 eV were recorded over a scan range of m/z 33–350. The linalool concentration was quantified using the linalool standard curve.

### Statistical analysis

Quantification of H_2_O_2_-containing areas and *Pst* growth were performed with the cellSens Entry software program (Olympus, Tokyo, Japan). The number of urediospore pustules was analyzed by ImageJ software. Statistical analyses of experimental groups including controls were evaluated by unpaired Student’s t-test in GraphPad Prism 8.

## Supplementary Information

Additional file 1: Supplementary Fig. S1. CRISPR-mediated gene editing of *TaWAKL8*-*2B.*

Additional file 2: Supplementary Fig. S2. Histological observations of fungal growth in *TaWAKL8-2B*-OE inoculated with *Pst* CYR32. A Histological observation of *Pst* growth and development. SV, substomatal vesicle; IH, infection hypha; HMC, haustorial mother cell; H, haustorium. B Hyphal length was measured by Cellsens software. Values represent the mean ± SEM of three independent samples with 90 infection sites. Asterisks indicate significant differences (Student’s *t* test, *p < 0.05, **p < 0.01, ***p < 0.001, ****p < 0.0001).

Additional file 3: Supplementary Fig. S3. DEGs in KEGG enrichment pathways. The majority of DEGs in KEGG enrichment pathways of 3 hpi (A) and 24 hpi (B) were down-regulated.

Additional file 4: Supplementary Table 1. Primers used in this study.

Additional file 5: Supplementary Table 2. DEGs identified in *TaWAKL8*-*2B*-KO transcriptome at 3 hpi and 24 hpi.

Additional file 6: Supplementary Table 3. DEGs identified in KEGG enrichment pathways at 3 hpi and 24 hpi.

## Data Availability

All data and materials are available in the paper and online supplemental files.

## Abbreviations

WAK: Wall-associated kinase
ROS: Reactive oxygen species
*Pst*: *Puccinia striiformis* F. sp. *tritici*
*Ptt*: *Puccinia triticina*
PTI: Pathogen-associated molecular pattern (PAMP)-triggered immunity
RLKs: Receptor-like kinases
RLPs: Receptor-like proteins
PRRs: Pattern recognition receptors
GUB: Galacturonan-binding
EGF: Epidermal growth factor
TM: Transmembrane
DEGs: Differentially expressed genes
LLS: Linalool synthase
POD: Peroxidase
SV: Substomatal vesicle
IH: Infection hypha
H, Haustorium (HMC): Haustorial mother cell
hpi: hours post inoculation
GC-MS: gas chromatography-mass spectrometer

## References

[CR1] Abbas F, Ke Y, Yu R, Yue Y, Amanullah S, Jahangir MM, Fan Y (2017) Volatile terpenoids: multiple functions, biosynthesis, modulation and manipulation by genetic engineering. Planta 246:803–816. 10.1007/s00425-017-2749-x 28803364 10.1007/s00425-017-2749-x

[CR2] Ahuja I, Kissen R, Bones A (2012) Phytoalexins in defense against pathogens. Trends Plant Sci 17:73–90. 10.1016/j.tplants.2011.11.002 22209038 10.1016/j.tplants.2011.11.002

[CR3] Almagro L, Gómez Ros LV, Belchi-Navarro S, Bru R, Ros Barceló A, Pedreño MA (2009) Class III peroxidases in plant defe-nce reactions. J Exp Bot 2:377–390. 10.1093/jxb/ern277 10.1093/jxb/ern277 19073963

[CR4] Anderson CM, Wagner TA, Perret M, He ZH, He D, Kohorn BD (2001) WAKs: cell wall-associated kinases li-nking the cytoplasm to the extracellular matrix. Plant Mol Biol 47:197–206. 10.1023/A:1010691701578 11554472

[CR5] Boutrot F, Zipfel C (2017) Function, Discovery, and exploitation of plant pattern recogni-tion receptors for broad-spectrum disease resistance. Annu Rev Phytopathol 55:257–286. 10.1146/annurev-phyto-080614-120106 28617654 10.1146/annurev-phyto-080614-120106

[CR6] Brutus A, Sicilia F, Macone A, Cervone F, De Lorenzo G (2010) A domain swap approach reveals a role of the plant wall-associated kinase 1 (WAK1) as a receptor of oligogalacturonides. Proc Natl Acad Sci U S A 107:9452–9457. 10.1073/pnas.1000675107 10.1073/pnas.1000675107 PMC2889104 20439716

[CR7] Chen X (2020) Pathogens which threaten food security: *Puccinia striiformis*, the wheat s-tripe rust pathogen. Food Secur 12:239–251. 10.1007/s12571-020-01016-z

[CR8] Chen F, Tholl D, Bohlmann J, Pichersky E (2011) The family of terpene synthases in plants: a mid-size family of genes for specialized metabolism that is highly di-versified throughout the kingdom. Plant J 66:212–229. 10.1111/j.1365-313X.2011.04520.x 21443633 10.1111/j.1365-313X.2011.04520.x

[CR9] Decreux A, Messiaen J (2005) Wall-associated kinase WAK1 interacts with cell wall pec-tins in a calcium-induced conformation. Plant Cell Physiol 46:268–278. 10.1093/pcp/pci026 15769808 10.1093/pcp/pci026

[CR10] DeFalco TA, Zipfel C (2021) Molecular mechanisms of early plant pattern-triggered immune signaling. Mol Cell 81:3449–3467. 10.1016/j.molcel.2021.07.029 34403694 10.1016/j.molcel.2021.07.029

[CR11] Delteil A, Gobbato E, Cayrol B, Estevan J, Michel-Romiti C, Dievart A, Kroj T, Morel JB (2016) Several wall-associated kinases participate positively and negatively in basal defense against rice blast fungus. BMC Plant Biol 16:17. 10.1186/s12870-016-0711-x 26772971 10.1186/s12870-016-0711-x PMC4715279

[CR12] Diener AC, Ausubel FM (2005) RESISTANCE TO FUSARIUM OXYSPORUM 1, a do-minant *Arabidopsis* disease-resistance gene, is not race specific. Genet 171:305–321. 10.1534/genetics.105.042218 10.1534/genetics.105.042218 PMC1456520 15965251

[CR13] Dmochowska-Boguta M, Kloc Y, Zielezinski A, Werecki P, Nadolska-Orczyk A, Karlowski WM, Orczyk W (2020) TaWAK6 encoding wall-asso-ciated kinase is involved in wheat resistance to leaf rust similar to adult plant resistance. PLoS ONE 15:e0227713. 10.1371/journal.pone.0227713 31929605 10.1371/journal.pone.0227713 PMC6957155

[CR14] Dumanović J, Nepovimova E, Natić M, Kuča K, Jaćević V (2021) The significance of reactive oxygenspecies and antioxidant defense system in plants: a concise overview. Front Plant Sci 11:552969. 10.3389/fpls.2020.552969 33488637 10.3389/fpls.2020.552969 PMC7815643

[CR15] Fang N, Jia C, Chen R, An J, Kang Z, Liu J (2024) The wheat CC-NBS-LRR protein TaRGA3 confersresistance to stripe rust by suppressing ascorbate peroxidase 6 activity. Plant Physiol 197:kiae603. 10.1093/plphys/kiae603 39556767 10.1093/plphys/kiae603

[CR16] Gao M, He Y, Yin X, Zhong X, Yan B, Wu Y, Chen J, Li X, Zhai K, Huang Y, Gong X, Chang H, Xie S, Liu J, Yue J, Xu J, Zhang G, Deng Y, Wang E (2021) Ca^2+^sensor-mediated ROS scavenging suppresses r-ice immunity and is exploited by a fungal effector. Cell 184:5391–5404. 10.1016/j.cell.2021.09.009 34597584 10.1016/j.cell.2021.09.009

[CR17] Gou JY, Li K, Wu K, Wang X, Lin H, Cantu D, Uauy C, Dobon-Alonso A, Midorikawa T, Inoue K, Sánchez J, Fu D, Blechl A, Wallington E, Fahima T, Meeta M, Epstein L, Dubcovsky J (2015) Wheat stripe rust resistance protein wks1 reduces the ability of the thylakoid-associated ascorbate peroxidase to detoxify reactive oxygen specie-s. Plant Cell 27:1755–1770. 10.1105/tpc.114.134296 25991734 10.1105/tpc.114.134296 PMC4498197

[CR18] Guo F, Wu T, Xu G, Qi H, Zhu X, Zhang Z (2021) TaWAK2A-800, a wall-associated kinase, particip-ates positively in resistance to *Fusarium* head blight and sharp eyespot in wheat. Int J Mol Sci 22:11493. 10.3390/ijms222111493 34768923 10.3390/ijms222111493 PMC8583783

[CR19] Harkenrider M, Sharma R, De Vleesschauwer D, Tsao L, Zhang X, Chern M, Canlas P, Zuo S, Ronald PC (2016) Overexpression of rice wall- associated kinase 25 (OsWAK25) alters resistance to bacterial and fungal pathogens. PLoS ONE 11:e0147310. 10.1371/journal.pone.0147310 26795719 10.1371/journal.pone.0147310 PMC4721673

[CR20] Hu K, Cao J, Zhang J, Xia F, Ke Y, Zhang H, Xie W, Liu H, Cui Y, Cao Y, Sun X, Xiao J, Li X, Zhang Q, Wang S (2017) Improvement of multiple agronomic traits by a disease resistance gene via cell wall reinforcement. Nat Plants 3:17009. 10.1038/nplants.2017.9 28211849 10.1038/nplants.2017.9

[CR21] Hu XH, Shen S, Wu JL, Liu J, Wang H, He JX et al (2023) A natural allele of proteasome maturation factor i-mproves rice resistance to multiple pathogens. Nat Plants 9:228–237. 10.1038/s41477-022-01327-3 36646829 10.1038/s41477-022-01327-3

[CR22] Huerta AI, Sancho-Andrés G, Montesinos JC, Silva-Navas J, Bassard S, Pau-Roblot C, Kesten C, Schlechter R, Dora S, Ayupov T, Pelloux J, Santiago J, Sánchez-Rodríguez C (2023) The WAK-like protein RFO1 acts as a sensor of the pectin methylation status in *Arabidopsis* cell walls to modulate r-oot growth and defense. Mol Plant 16:865–881. 10.1016/j.molp.2023.03.015 37002606 10.1016/j.molp.2023.03.015 PMC10168605

[CR23] Huluka W, Kumsa L (2022) Analysis of rice (Oryza sativa L. ssp. Japonica) wall assoc-iated receptor-like protein kinase gene’s promoter region and regulatory elements. Curr Pl-ant Biol 31:100254. 10.1016/j.cpb.2022.100254

[CR24] Ishida Y, Tsunashima M, Hiei Y, Komari Tl (2015) Wheat (*Triticum aestivum L.*) Transformati-on Using Immature Embryos. Methods Mol Biol 1223:189–198. 10.1007/978-1-4939-1695-5_15 25300841 10.1007/978-1-4939-1695-5_15

[CR25] Jiang Y, Pan X, Li Y, Yang Y, Jia Y, Lei B, Feng J, Ma Z, Liu X, Yan H (2023) Linalool induces resistance against tobacco mosaic virus in tobacco plants. Plant Dis 107:2144–2152. 10.1094/PDIS-09-22-2246-RE 36917091 10.1094/PDIS-09-22-2246-RE

[CR26] Khan MH, Bukhari A, Dar ZA, Rizvi SM (2013) Status and strategies in breeding for rust resistance in wheat. Agricul Sci 4:292–301. 10.4236/as.2013.46042

[CR27] King J, Dreisigacker S, Reynolds M, Bandyopadhyay A, Braun HJ, Crespo-Herrera L et al (2024) Wheat genetic resources have avoided disease pandemics, improved food security, and reduced environmental footprints: a revie-w of historical impacts and future opportunities. Glob Change Biol 30:ce17440. 10.1111/gcb.17440 10.1111/gcb.17440 39185562

[CR28] Kohorn BD (2015) Cell wall-associated kinases and pectin perception. J Exp Bot 67:489–494. 10.1093/jxb/erv467 26507892 10.1093/jxb/erv467

[CR29] Kohorn BD, Kohorn SL (2012) The cell wall-associated kinases, WAKs, as pectin receptors. Front Plant Sci 3:88. 10.3389/fpls.2012.00088 22639672 10.3389/fpls.2012.00088 PMC3355716

[CR30] Kohorn BD, Johansen S, Shishido A, Todorova T, Martinez R, Defeo E, Obregon P (2009) Pectin activation of MAP kinase and gene expression is WAK2 dependent. Plant J 60:974–982. 10.1111/j.1365-313X.2009.04016.x 19737363 10.1111/j.1365-313X.2009.04016.x PMC3575133

[CR31] Laupheimer S, Ghirardo A, Kurzweil L, Weber B, Stark TD, Dawid C, Schnitzler JP, Hückelhoven R (2024) *Blumeria hordei* affects volatile emission of susceptible and resistant barley plants and modifies the defense response of recipient plants. Physiol Planta 176:e14646. 10.1111/ppl.14646 10.1111/ppl.14646 PMC11626344 39648862

[CR32] Li H, Zhou SY, Zhao WS, Su SC, Peng YL (2009) A novel wall-associated receptor-like protein kin-ase gene, OsWAK1, plays important roles in rice blast disease resistance. Plant Mol Biol 69:337–346. 10.1007/s11103-008-9430-5 19039666 10.1007/s11103-008-9430-5

[CR33] Li W, Shao M, Yang J, Zhong W, Okada K, Yamane H, Qian G, Liu F (2013) OsCYP71Z2 involves diterpenoid phytoalexin biosynthesis that contributes to bacterial blight resistance in rice. Plant Sci 207:98–107. 10.1016/j.plantsci.2013.02.005 23602104 10.1016/j.plantsci.2013.02.005

[CR34] Li W, Zhu Z, Chern M, Yin J, Yang C, Ran L et al (2017) A natural allele of a transcription factor in rice confers broad-spectrum blast resistance. Cell 170:114–126. 10.1016/j.cell.2017.06.008 28666113 10.1016/j.cell.2017.06.008

[CR35] Li F, Li W, Lin YJ, Pickett JA, Birkett MA, Wu K, Wang G, Zhou JJ (2018) Expression of lima bean terpene synthases in rice en-hances recruitment of a beneficial enemy of a major rice pest. Plant Cell Environ 41:111–120. 10.1111/pce.12959 28370092 10.1111/pce.12959

[CR36] Li Q, Hu A, Qi J, Dou W, Qin X, Zou X, Xu L, Chen S, He Y (2020) CsWAKL08, a pathogen-induced wall-associated receptor-like kinase in sweet orange, confers resistance to citrus bacterial canker via ROS control and JA signaling. Hortic Res 7:42. 10.1038/s41438-020-0263-y 32257228 10.1038/s41438-020-0263-y PMC7109087

[CR37] Liang J, Shen Q, Wang L, Liu J, Fu J, Zhao L, Xu M, Peters RJ, Wang Q (2021) Rice contains a biosynthetic gene cluster associated with production of the casbane-type diterpenoid phytoalexin ent-10-oxodepressin. New Phytol 231:85–93. 10.1111/nph.17406 10.1111/nph.17406 PMC9044444 33892515

[CR38] Liu Z, Bowden RL, Bai G (2013) Molecular markers for leaf rust resistance gene Lr42 in wheat. Crop Sci 53:1566–1570. 10.2135/cropsci2012.09.0532

[CR39] Liu Q, Wang C, Jiao X, Zhang H, Song L, Li Y, Gao C, Wang K (2019) Hi-TOM: a platform for high-throughput tracking of mutations induced by CRISPR/Cas systems. Sci China Life Sci 62:1–7. 10.1007/s11427-018-9402-9 30446870 10.1007/s11427-018-9402-9

[CR40] Liu X, Yan W, Liu S, Wu J, Leng P, Hu Z (2024) LiNAC100 contributes to linalool biosynthesis by directly regulating LiLiS in *Lilium* “Siberia”. Planta 259:73. 10.1007/s00425-024-04340-2 38393405 10.1007/s00425-024-04340-2

[CR41] Liu Z, Zhang Z, Faris JD, Oliver RP, Syme R, McDonald MC, McDonald BA, Solomon PS, Lu S, Shelver WL, Xu S, Friesen TL (2012) The cysteine rich necrotrophic effector SnTox1 produced by *Stagonospora nodorum* triggers susceptibility of wheat lines harboring Snn1. Plos Pathog 8:e1002467. 10.1371/journal.ppat.1002467 10.1371/journal.ppat.1002467 PMC3252377 22241993

[CR42] Lu X, Zhang J, Brown B, Li R, Rodríguez-Romero J, Berasategui A, Liu B, Xu M, Luo D, Pan Z, Baerson SR, Gershenzon J, Li Z, Sesma A, Yang B, Peters RJ (2018) Inferring Roles in Defense from Metabolic Allocation of Rice Diterpenoids. Plant Cell 30:1119–1131. 10.1105/tpc.18.00205 29691314 10.1105/tpc.18.00205 PMC6002189

[CR43] Macho AP, Zipfel C (2014) Plant PRRs and the activation of innate immune signaling. Mol Cell 54:263–272. 10.1016/j.molcel.2014.03.028 24766890 10.1016/j.molcel.2014.03.028

[CR44] Malukani KK, Ranjan A, Hota SJ, Patel HK, Sonti RV (2020) Dual activities of receptor-like kinase O-sWAKL21.2 induce immune responses. Plant Physiol 183:1345–1363. 10.1104/pp.19.01579 32354878 10.1104/pp.19.01579 PMC7333719

[CR45] Mittler R, Zandalinas SI, Fichman Y, Van Breusegem F (2022) Reactive oxygen species signaling in plant stress responses. Nat Rev Mol Cell Biol 23:663–679. 10.1038/s41580-022-00499-2 35760900 10.1038/s41580-022-00499-2

[CR46] Ngou B, Jones J, Ding PT (2021) Plant immune networks. Trends Plant Sci 27:255–273. 10.1016/j.tplants.2021.08.012 34548213 10.1016/j.tplants.2021.08.012

[CR47] Pfaffl MW (2001) A new mathematical model for relative quantification in real-time RT-PCR. Nucleic Acids Res 29:e45. 10.1093/nar/29.9.e45 11328886 10.1093/nar/29.9.e45 PMC55695

[CR48] Polturak G, Dippe M, Stephenson MJ, Chandra Misra R, Owen C, Ramirez-Gonzalez RH, Haidoulis JF, Schoonbeek HJ, Chartrain L, Borrill P, Nelson DR, Brown JKM, Nicholson P, Uauy C, Osbourn A (2022) Pathogen-induced biosynthetic pathwaysencode defense-related molecules in bread wheat. Proc Natl Acad Sci USA 119:e2123299119. 10.1073/pnas.2123299119 35412884 10.1073/pnas.2123299119 PMC9169793

[CR49] Qi J, Wang J, Gong Z, Zhou JM (2017) Apoplastic ROS signaling in plant immunity. Curr Opin Plant Biol 38:92–100. 10.1016/j.pbi.2017.04.022 28511115 10.1016/j.pbi.2017.04.022

[CR50] Qi H, Guo F, Lv L, Zhu X, Zhang L, Yu J, Wei X, Zhang Z (2021) The wheat wall-associated receptor-like kinase TaWAK-6D mediates broad resistance to two fungal pathogens *Fusarium pseudograminearum* and *Rhizoctonia cerealis*. Front in Plant Sci 12:2322. 10.3389/fpls.2021.758196 10.3389/fpls.2021.758196 PMC8579037 34777437

[CR51] Saintenac C, Lee WS, Cambon F, Rudd JJ, King RC, Marande W, Powers SJ, Bergès H, Phillips AL, Uauy C, Hammond-Kosack KE, Langin T, Kanyuka K (2018) Wheat receptor-kinase-like protein Stb6 controls gene-for-gene resistance to fungal pathogen *Zymoseptoria tritici*. Nat Genet 50:368–374. 10.1038/s41588-018-0051-x 29434355 10.1038/s41588-018-0051-x

[CR52] Savary S, Willocquet L, Pethybridge SJ, Esker P, McRoberts N, Nelson A (2019) The global burden of pathogens andpests on major food crops. Nat Ecol Evol 3:430–439. 10.1038/s41559-018-0793-y 30718852 10.1038/s41559-018-0793-y

[CR53] Shi G, Zhang Z, Friesen TL, Raats D, Fahima T, Brueggeman RS, Lu S, Trick HN, Liu Z, Chao W, Frenkel Z, Xu SS, Rasmussen JB, Faris JD (2016) The hijacking of a receptor kinas driven path-way by a wheat fungal pathogen leads to disease. Sci Adv 2:e1600822. 10.1016/j.pbi.2020.04.003 10.1126/sciadv.1600822 PMC5091353 27819043

[CR54] Stephens C, Hammond-Kosack KE, Kanyuka K (2022) WAKsing plant immunity, waning diseases. J Exp Bot 73:22–37. 10.1093/jxb/erab422 34520537 10.1093/jxb/erab422

[CR55] Taniguchi S, Hosokawa-Shinonaga Y, Tamaoki D, Yamada S, Akimitsu K, Gomi K (2014) Jasmonate induction of the monoterpene linalool confers resistance to rice bacterial blight and its biosynthesis is reg-ulated by JAZ protein in rice. Plant Cell Environ 37:451–461. 10.1111/pce.12169 23889289 10.1111/pce.12169

[CR56] Verica JA, He ZH (2002) The cell wall-associated kinase (WAK) and WAK-like kinase gene family. Plant Physiol 129:455–459. 10.1104/pp.011028 12068092 10.1104/pp.011028 PMC1540232

[CR57] Wagner TA, Kohorn BD (2001) Wall-associated kinases are expressed throughout plant d-evelopment and are required for cell expansion. Plant Cell 13:303–318. 10.1105/tpc.13.2.303 11226187 10.1105/tpc.13.2.303 PMC102244

[CR58] Wang CF, Huang LL, Buchenauer H, Han QM, Zhang HC, Kang ZS (2007) Histochemical studies on the accumula-tion of reactive oxygen species (O^2-^ and H_2_O_2_) in the incompatible and compatible inter-action of wheat-*Puccinia striiformis* f. sp. *tritici*. Physiol Mol Plant Pathol 71:230–239. 10.1016/j.pmpp.2008.02.006

[CR59] Wang L, Xu G, Li L, Ruan M, Bennion A, Wang GL, Li R, Qu S (2023a) The OsBDR1-MPK3 module negatively regulates blast resistance by suppressing the jasmonate signaling and terpenoid biosynthesis pathway. Proc Natl Acad Sci U S A 120:e2211102120. 10.1073/pnas.2211102120 36952381 10.1073/pnas.2211102120 PMC10068787

[CR60] Wang Q, Wang X, Huang L, Cheng Y, Ren L, Yang H, Zhou C, Wang X, He J (2023b) Promoter characterization of a citrus lina-lool synthase gene mediating interspecific variation in resistance to a bacterial pathogen. BMC Plant Biol 23:405. 10.1186/s12870-023-04413-6 37620808 10.1186/s12870-023-04413-6 PMC10463377

[CR61] Wu B, Qi F, Liang Y (2023) Fuels for ROS signaling in plant immunity. Trends in Plant Sci 28:1124–1131. 10.1016/j.tplants.2023.04.007 37188557 10.1016/j.tplants.2023.04.007

[CR62] Xu Q, Tang C, Wang X, Sun S, Zhao J, Kang Z, Wang X (2019) An effector protein of the wheat stripe rust fu-ngus targets chloroplasts and suppresses chloroplast function. Nat Commun 10:5571. 10.1038/s41467-019-13487-6 31804478 10.1038/s41467-019-13487-6 PMC6895047

[CR63] Yang P, Praz C, Li B, Singla J, Robert CAM, Kessel B, Scheuermann D, Lüthi L, Ouzunova M, Erb M, Krattinger SG, Keller B (2019) Fungal resistance mediated by maize wall-associated kinase ZmWAK-RLK1 correlates with reduced benzoxazinoid content. New Phytol 221:976–987. 10.1111/nph.15419 30178602 10.1111/nph.15419

[CR64] Zhan C, Lei L, Liu Z, Zhou S, Yang C, Zhu X et al (2020) Selection of a subspecies-specific diterpene gene cluster implicated in rice disease resistance. Nat Plants 6:1447–1454. 10.1038/s41477-020-00816-7 33299150 10.1038/s41477-020-00816-7

[CR66] Zhang Q, Xu Q, Zhang N, Zhong T, Xing Y, Fan Z, Yan M, Xu M (2024) A maize WAK-SnRK1α2-WRKY module regulates nutrient availability to defend against head smut disease. Mol Plant 17:1654–1671. 10.1016/j.molp.2024.09.013 39360383 10.1016/j.molp.2024.09.013

[CR67] Zhao N, Guo A, Wang W, Li B, Wang M, Zhou Z, Jiang K, Aierxi A, Wang B, Adjibolosoo D, Xia Z, Li H, Cui Y, Kong J, Hua J (2024) GbPP2C80 Interacts with GbWAKL14 to negatevely co-regulate resistance to *Fusarium* and *Verticillium* wilt via MPK3and ROS signalingin Sea Island Cotton. Adv Sci 11:e2309785. 10.1002/advs.202309785 38889299 10.1002/advs.202309785 PMC11321686

[CR68] Zhong T, Zhu M, Zhang Q, Zhang Y, Deng S, Guo C, Xu L, Liu T, Li Y, Bi Y, Fan X, Balint-Kurti P, Xu M (2024) The ZmWAKL-ZmWIK-ZmBLK1-ZmRBOH4 module provides quantitative resistance to gray leaf spot in maize. Nat Genet 56:315–326. 10.1038/s41588-023-01644-z 38238629 10.1038/s41588-023-01644-z PMC10864183

[CR69] Zuo N, Bai WZ, Wei WQ, Yuan TL, Zhang D, Wang YZ, Tang WH (2022) Fungal CFEM effectors negatively regulate a maize wall-associated kinase by interacting with its alternatively spliced variant to dampen resistance. Cell Rep 41:111877. 10.1016/j.celrep.2022.111877 36577386 10.1016/j.celrep.2022.111877

