# Supplementary figures and images for: TaWAKL8-2B, a wall-associated receptor-like kinase, mediates wheat rust resistance by linalool and ROS accumulation
Source: Stress Biol. 2025 Aug 18;5(1):50. doi: 10.1007/s44154-025-00248-3 (PMC12361011; doi:10.1007/s44154-025-00248-3)

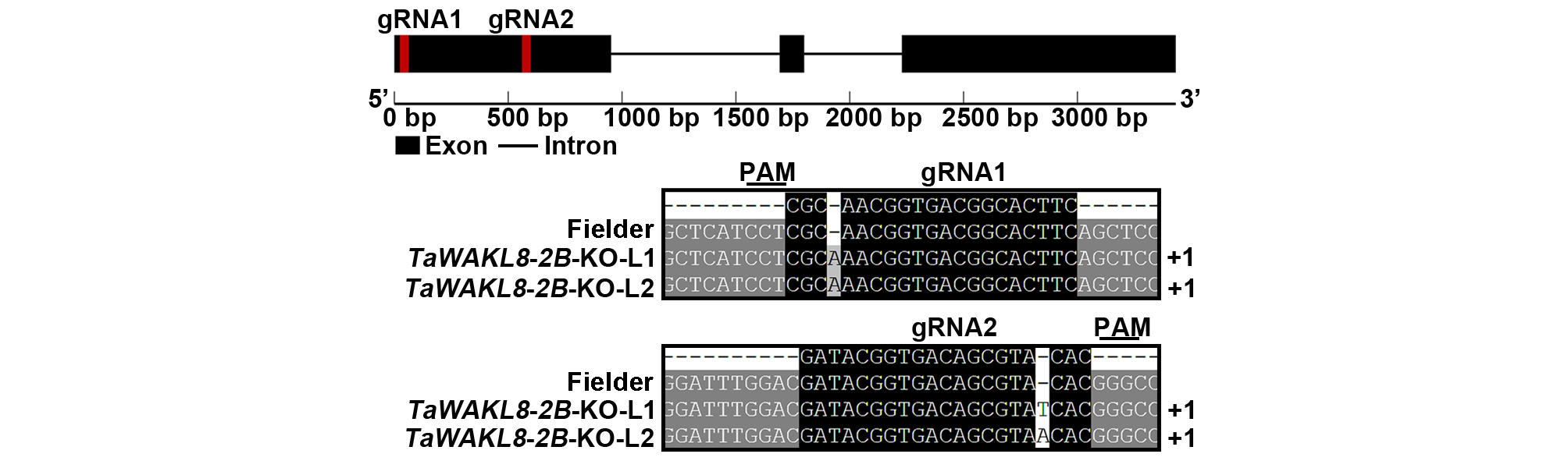

Supplement: Supplementary file 1 — Additional file 1: Supplementary Fig. S1. CRISPR-mediated gene editing of TaWAKL8-2B. [file 44154_2025_248_MOESM1_ESM.tif]

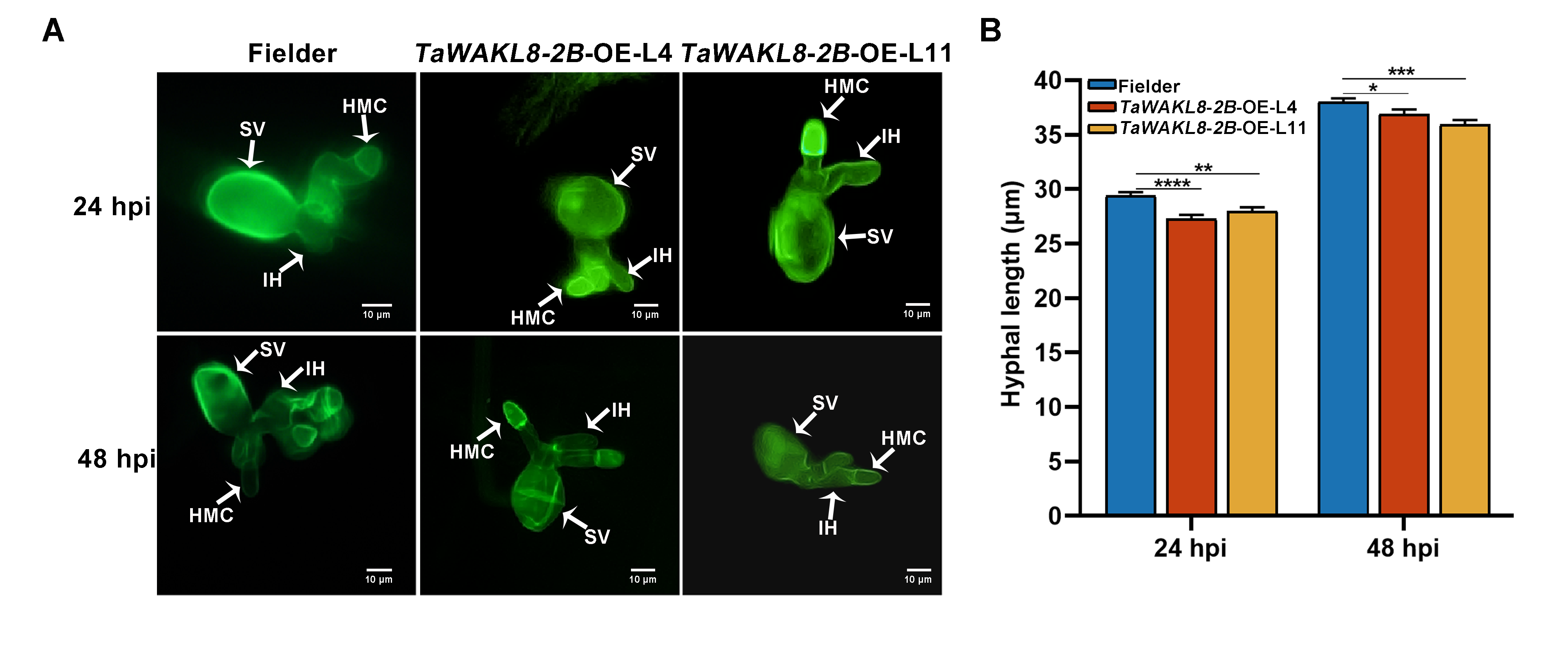

Supplement: Supplementary file 2 — Additional file 2: Supplementary Fig. S2. Histological observations of fungal growth in TaWAKL8-2B-OE inoculated with Pst CYR32. A Histological observation of Pst growth and development. SV, substomatal vesicle; IH, infection hypha; HMC, haustorial mother cell; H, haustorium. B Hyphal length was measured by Cellsens software. Values represent the mean ± SEM of three independent samples with 90 infection sites. Asterisks indicate significant differences (Student’s t test, *p < 0.05, **p < 0.01, ***p < 0.001, ****p < 0.0001). [file 44154_2025_248_MOESM2_ESM.tif]

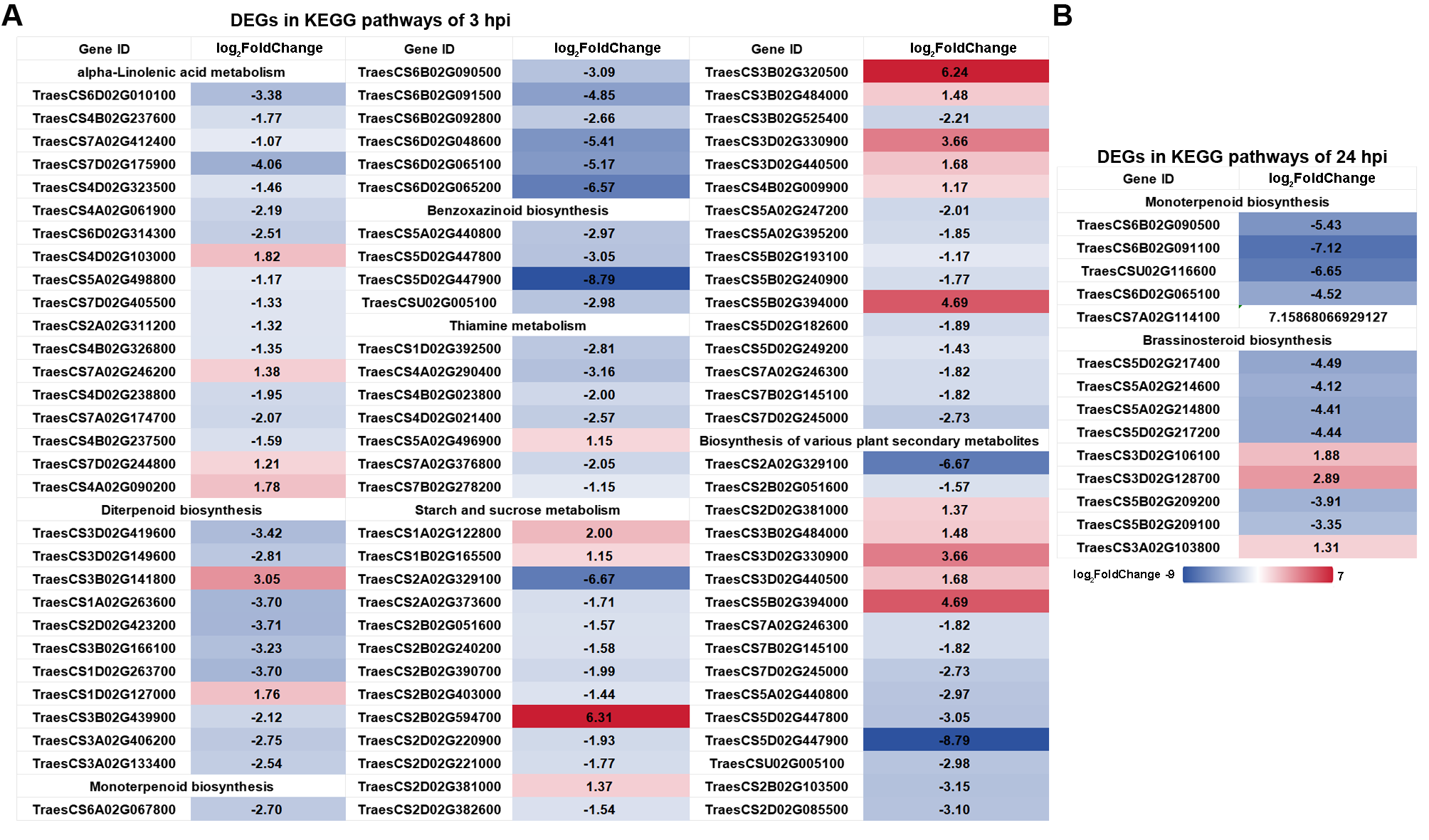

Supplement: Supplementary file 3 — Additional file 3: Supplementary Fig. S3. DEGs in KEGG enrichment pathways. The majority of DEGs in KEGG enrichment pathways of 3 hpi (A) and 24 hpi (B) were down-regulated. [file 44154_2025_248_MOESM3_ESM.tif]
